# Seasonal forecasting of the hourly electricity demand applying machine and deep learning algorithms impact analysis of different factors

**DOI:** 10.1038/s41598-025-91878-0

**Published:** 2025-03-18

**Authors:** Heba-Allah Ibrahim El-Azab, R. A. Swief, Noha H. El-Amary, H. K. Temraz

**Affiliations:** 1 Faculty of Engineering, Ahram Canadian University (ACU), Giza, Egypt; 2 Faculty of Engineering, Ain Shams University, Cairo, 11517 Egypt; 3 Vice President for Community Service and Environmental Development Affairs, Sixth of October University (O6U), Giza, Egypt; 4 Arab Academy for Science, Technology and Maritime Transport (AASTMT), Cairo, 2033 Egypt

**Keywords:** Hourly demand forecasting, Temperature, Electricity price, Day-Type, Gated recurrent units, Long short-term memory, Adaptive neuro-fuzzy inference system, Time-series forecasting, Mean absolute percentage error, Engineering, Energy grids and networks, Power distribution

## Abstract

The purpose of this paper is to suggest short-term Seasonal forecasting for hourly electricity demand in the New England Control Area (ISO-NE-CA). Precision improvements are also considered when creating a model. Where the whole database is split into four seasons based on demand patterns. This article’s integrated model is built on techniques for machine and deep learning methods: Adaptive Neural-based Fuzzy Inference System, Long Short-Term Memory, Gated Recurrent Units, and Artificial Neural Networks. The linear relationship between temperature and electricity consumption makes the relationship noteworthy. Comparing the temperature effect in a working day and a temperature effect on a weekend day where at night, the marginal effects of temperature on the demand in a working day for power are likewise at their highest. However, there are significant effects of temperature on the demand for a holiday, even a weekend or special holiday. Two scenarios are used to get the results by using machine and deep learning techniques in four seasons. The first scenario is to forecast a working day, and the second scenario is to forecast a holiday (weekend or special holiday) under the effect of the temperature in each of the four seasons and the cost of electricity. To clarify the four techniques’ performance and effectiveness, the results were compared using the Mean Absolute Error (MAE), Root Mean Squared Error (RMSE), Normalized Root Mean Squared Error (NRMSE), and Mean Absolute Percentage Error (MAPE) values. The forecasting model shows that the four highlighted algorithms perform well with minimal inaccuracy. Where the highest and the lowest accuracy for the first scenario are (99.90%) in the winter by simulating an Adaptive Neural-based Fuzzy Inference System and (70.20%) in the autumn by simulating Artificial Neural Network. For the second scenario, the highest and the lowest accuracy are (96.50%) in the autumn by simulating Adaptive Neural-based Fuzzy Inference System and (68.40%) in the spring by simulating Long Short-Term Memory. In addition, the highest and the lowest values of Mean Absolute Error (MAE) for the first scenario are (46.6514, and 24.759 MWh) in the spring, and the summer by simulating Artificial Neural Networks. The highest and the lowest values of Mean Absolute Error (MAE) for the second scenario are (190.880, and 45.945 MWh) in the winter, and the autumn by simulating Long Short-Term Memory, and Adaptive Neural-based Fuzzy Inference System.

## Introduction

Predicting the short-term load of electricity demand becomes a critical challenge for energy providers, system administrators, and other market stakeholders. Worldwide, power demand is determined by hourly bidding for a single day earlier than delivery occurs in numerous deregulated marketplaces for electrical power. Increased demand prediction accuracy can reduce operating costs and increase electrical power dependability. Developing reliable models is challenging due to the complex nature of power consumption. Enough electricity is required for intelligent systems, such as the Internet of Things (IoT), to link " whatever from wherever"^1^.

According to the United Nations Commission on Global Weather Change’s fifth annual report, global warming has led to an increase in the world temperature by almost 0.74 °C, and it is expected to reach 1.8–4 °C by the end of the twenty-first century. The widespread installation of cooling (AC) units has caused the highest-demand events to move from the end of the day to daylight hours because of the rise in temperatures^2,3^. In addition, the widespread usage of air conditioning and heating appliances, which are mostly utilized by homeowners and companies in domestic, industrial, and commercial sectors, is generally associated with this impact^4^. In residential structures, the energy consumed by air conditioning, heating, and ventilation systems is greater than 50%^5^. Determined factors like day kinds and seasonal months also demonstrate their effects on demand, throughout the temperature. Demand patterns are strongly influenced by the industries that utilize electrical power^6^. The impact that household lifestyle has on energy consumption is becoming more and more notable. Effective power governance requires strict control over end-user demand because electricity cannot be stored efficiently^7^. To ensure the stability of the energy system while it is working and to retain a safe, sufficient, and effective availability of electrical power by lowering the danger of a blackout, The core objective of this manuscript is to develop a highly precise forecasting framework and conduct a quantitative investigation of the demand variables that influence it.

Therefore, to ensure dependability and continuation of improved power performance in the electrical power grid, anticipating the price of electricity requires both accuracy and stability^8^. The following are some of the main variables that affect how much electricity is consumed fluctuating: temperature effect, humidity, wind speed, cloud cover, intensity of daily activities, working and non-working days, special holidays, fuel prices, etc. These variables lead to price volatility and non-linearity. The energy market operators have to deal with the aforementioned problems as a result of the dynamic power price’s unpredictability and volatility^9,10^. To anticipate the price of energy, several elements, hourly demand and cost of electricity signals, for example, ought to be able to be expressed as inputs^11^.

While certain predictable patterns, like seasonal, weekly, and daily trends, are present, actual electricity usage varies as a result of consumers’ erratic habits. Lifecycle behaviors are brought about by variations in demand in the morning, afternoon, and evening. The researcher has two options for dealing with these during-the-day periodicities: first, assign each hour’s or half-hour’s worth of variables individually. The second approach involves creating distinct models for every hour or half-hour to eliminate throughout-the-day seasonality^12–15^. In the same way, working and days off account for the seasonality of weekdays. A comparable practice with separate factors for every single day utilizing fictitious variables is used to eliminate such seasonality. The varying temperature in each season causes annual seasonality, which is eliminated by using fictitious variables. The fictitious factors additionally handle seasonality in each month^14,16^.

Most recent studies have been transformed by techniques based on machine learning and deep learning, which produce dependable and precise forecasting results^17^. In the electricity sector, time-series approaches that describe seasonality and patterns of historical data, such as seasonal autoregressive integrated moving average (SARIMA) or autoregressive integrated moving average model (ARIMA), have been extensively applied for electric power forecasting^18–20^. One disadvantage of statistical approaches, which are usually linear and skilled in prediction, is that they may not work well with highly variable data, like hourly information at a rapid rate. Examples of such techniques include multiple linear regression, exponential smoothing, autoregressive moving average (ARMA), autoregressive integrated moving average (ARIMA), and vector autoregression (VAR). Specifically, hourly pricing’s unstable trends, which work well with low data frequency like weekly themes, may become too complicated for forecasting purposes^21,22^. In^23,24^ by using the advantages of many methods to improve the precision of predictions, mixed models that include several methods for forecasting have also demonstrated effectiveness. Consequently, dealing with time-series data presents several difficult challenges, investigators have turned to Artificial Neural Networks (ANN), which are modeled after the framework of the brain of humans. They have proven to be exceptionally accomplished in many different types of fields, including Natural Language Processing (NLP), audio identification, medicine, and load forecasting. The requirement for massive amounts of data to train ANN models is one of their difficult issues^25–28^.

Machine and deep learning models both incorporate intelligence-based approaches. Support vector machines and artificial neural-based networks are examples of machine learning approaches. However, Deep learning approaches have improved precision, efficacy, and outcomes during the past few years. The fast growth of efficient hardware for computing and useful applications has prompted the usage of advanced machine and deep learning techniques. Consequently, the precision and power of employing models based on machine learning and deep learning, and a mix of both, has led to the construction of a forecasting model with an intricate network topology. Deep learning methods include convolutional neural networks (CNN) with the immunity of the lion approach, long-short term memory (LSTM), gated recurrent units (GRU), and recurrent neural networks (RNN). ANFIS, fuzzy + neural network, ANN, SVM, and genetic algorithm (GA) are examples of hybrid techniques^29–32^.

### An overview of relevant literature and history

Song et al.^33^ proposed in their studies in Bangladesh, that months of summer and wintertime months experience significant temperature variations. However, because of high temperatures throughout the summer, there is a large rise in the requirement for electrical load because of the frequent usage of air-cooling units. It is significant to remember that load prediction precision decreases in both two seasons when the relationship between load and temperature is not taken into account. By using the temperature-sensitive nature of demand in load forecasting, this issue can be resolved^33^.

The article in^34^ provided a method for analyzing the effects of global warming on the daily peak load using a linear regression model. Lastly, a three-point technique was applied to predict the temperature responsiveness of the load based on the outcomes. It had been noted that temperature had a noteworthy influence on the load pattern. The temperature impact of the load was predicted considering the historical data. All additional weather variables, such as moisture, wind speed, and cloud cover, can also be included in this study. According to the authors, the prediction of electrical consumption and temperature sensitivity would be highly useful in incorporating climate factors into electric power system design^34^.

The growing prevalence of smart grids has made forecasting future loads extremely important. Some variables, including weather conditions, could have an impact on the results when predicting future load consumption. The absence of upcoming weather has presented a difficult issue for load forecasting. This article has discussed the past demand as a predictor to estimate demand for one step forward. Certain non-deep learning techniques, such as ARIMA or linear regression, were effective methods for producing precise load forecasts. However, regression-based techniques do have certain drawbacks. By using autocorrelation (AC) quantities, lags are employed as variables in techniques like Support Vector Regression and regression with linearity. The number of lags as variables of regression systems might vary as a cutoff value is indeterminate. More inaccuracies could result from this process (identifying lags by autocorrelation graph). Two other recognized time-series study methods are Exponential Smoothing (ETS) and ARIMA. However, for these techniques to function, a few parameters must be adjusted. To figure out the most acceptable possible values for them, this technique requires several attempts. In addition, data analysis is required for time-series approaches to determine whether or not data are constant. On the other hand, even if the data was fixed, LSTM could still produce good results. Another hybrid model was employed in several load forecasting investigations by CNN-LSTM^35^.

Lopez et al.^36^ proposed a system’s load series that exhibited recurring trends on a daily, weekly, and annual basis. However, irregularities in this general periodic activity are brought on by other circumstances, such as social gatherings or temperature. Understanding and modeling these variations was essential to creating a load forecasting system that works, as a significant portion of load forecasting losses are frequently associated with the increased forecasting inaccuracy typical of these unique days. The impact of various special day kinds on the load distribution curve was the main topic of this research, along with the significance of accurately modeling these behaviors. This study modeled the impact of social occurrences, such as holidays or festive times, on the load distribution curve using linear regression and examined the Spanish national network. The findings in this research demonstrated that a comprehensive categorization of occurrences was required to precisely model every possible event during a seven-year timeframe. The primary inferences that can be made from this were that the mean error for special days produced by the suggested special day categorization was 1.84%, which was extremely close to the mean error for normal days which was estimated by 1.78%. Furthermore, in model 7, the 95th percentile for special days was a mere 4.56%, down from 17.6% in model 0. This indicated that the simulation error for just 5% of exceptional days was greater than 4.56%. On ordinary days, the percentage was significantly lower estimated at 4.33%^36^.

Srinivasan et al.^37^ presented in this study, the execution and prediction outcomes of a hybrid fuzzy neural technique for power demand prediction, which blends fuzzy logic and the theory of fuzzy sets algorithms with neural network simulation. This potent technique’s capabilities were found in its capacity to predict with accuracy not only on weekends and public holidays but also on weekdays and days leading up to and following them. Fuzzy logic was also a useful tool for handling load changes brought on by unusual events. A fuzzy neural network (FNN) for 24-h forward forecasting powered by anticipated weather data had been thoroughly evaluated using real data taken from a power system. The average inaccuracy on weekdays was 0.62%, on Saturdays it was 0.83%, and on Sundays and public holidays, it was 1.17%^37^.

Ziel et al.^38^ discussed how to predict electricity demand during public or bank holidays. Specific features of public holidays, including their division into weekday and fixed-date holidays, were covered in depth. This study offered cutting-edge methods for handling public holidays, including their removal from the data set, treatment as Sunday dummies, and introduction of distinct holiday dummies. The study weighed the benefits and drawbacks of each strategy and presented a comprehensive load forecasting analysis for Germany that contrasts the methods based on accepted performance and significance metrics. The study offered broad guidelines for handling public holidays concerning electric power forecasting, highlighting specific ways to reduce the shortcomings of the majority of cutting-edge techniques. This was particularly helpful since it could increase the precision of forecasting by over 80% during public holiday periods. Even during non-holidays at certain times, the forecast error can be decreased by about 10%. The experimental outcomes demonstrated that adding holiday impacts could significantly increase predicting performance. The enhancement could reach up to 80% on public holidays, although interestingly, the precision of the forecast rises by roughly 10% even during non-holiday times owing to well-covered holiday effects. The public holiday fictitious approach was the most feasible strategy for handling public holidays. This methodology was particularly effective in modeling with multiple variables contexts. The workday dummies during the holidays were set to zero in the switching public holiday dummy strategy, but public holiday dummies were added to the framework as well^38^.

Lusis et al.^39^ examined the relationship between the length of the training set, forecasting precision, and calendar influences on the accuracy of a day-ahead load estimate for consumers in residential areas. The forecast error metrics that were measured were root mean square error (RMSE) and normalized RMSE. While the average RMSE results from regression trees, neural networks, and support vector regression were comparable, the statistical evaluation revealed that the regression trees approach was noticeably superior. The apparent calendar impacts had minimal prediction ability when daily and weekly seasonality in historical load profiles were supplemented with meteorological information. Using a finer prediction precision was found to decrease prediction errors in the simulation under study. Additionally, it was discovered that a load forecast model for residential users might be developed using just a year’s worth of information from past records, with the negligible effect of increasing the dataset used for training. For most of the scenarios in this study, it was found that the addition of binary variables representing calendar effects produced a lower error than the subsets strategy. However, static analysis failed to find a statistically significant difference between the scenarios with and without calendar impacts, indicating that calendar impacts were not repetitive. A smoother load profile from a considerably higher number of residences would render periodic load trends (intra-day, weekly, and seasonal) more pronounced and predictable^39^.

This research^40^ offered a thorough examination of numerous cutting-edge techniques for objective and deterministic LV demand predictions. Hapen et al. assessed the prediction accuracy of these out-of-sample approaches for up to four days ahead of time on 100 actual LV feeders. Furthermore, they investigated how the temperature—both real and predicted—affected the demand precision estimates. They also gave some significant findings regarding the factors that influenced forecast accuracy that relied on the empirical comparison of forecast indicators that are uncertain and point-based. Since the standards were set a full week in advance, they utilized each seven in the morning as the forecast starting point in the sampled-post information to maintain consistency with the temperature-predicted data from the observatory. As anticipated, the most accurate forecasts were a single day ahead of time, while the least precise predictions occurred for four days onward. It was found that a Strong correlation exists between the errors which was nearly 0.995. Furthermore, a minimum group of errors was obtained by using mean absolute percentage error (MAPE) and Root Mean Square Error (RMSE).

According to this analysis^41^, one of the most appealing stochastic alternative energy sources that lower greenhouse gas emissions is the development of battery-powered automobiles. The nature of seasonal influence has been investigated using four different forecasting models. Four distinct forecasting networks have been developed in order to increase the system’s accuracy and gain a better understanding of how seasonal elements, such as temperature differences over the four seasons, influence the battery of electric vehicles in both the charging and discharging phases. These factors affect how accurate the forecasting model is. Four featured algorithms are examined. Deep learning techniques like Long Short-Term Memory and Gated Recurrent Units are covered, in addition to machine learning techniques like Artificial Neural Networks and Adaptive Neuro-Fuzzy Inference Systems. The Gated Recurrent Units network replicates somewhat better on an hourly basis median every day of the previous records of charging battery-powered cars than the long short-term memory network. The advantages of neural network technology and the fuzzy inference network are combined in the Adaptive Neuro-Fuzzy Inference System. Because deep learning technology is used, the projected outcome generated by the Gated Recurrent Unit technique is more accurate and has a smaller mean absolute percentage error (MAPE) than the outcomes produced by the Long Short-Term Memory algorithm. This is because, with a precision of 99.3%, 97.7%, and 98.3%, the total MAPEs in the 24-h period have decreased by 0.1203%, 0.2397%, and 0.0735% throughout the winter, spring, and summer, respectively. However, with a 98.08% precision, the overall MAPE increased by 0.2253% throughout the fall season. The expected dataset from the adaptive neuro-fuzzy inference is more accurate with a less mean absolute percentage error than the results of the neural network modeling method. This is because, with an accuracy of 99.35%, 98.04%, 98.56, and 97.06%, the total MAPEs in the 24 h have decreased by 6.1907%, 1.2103%, 0.8812, and 0.7236% in the winter, spring, and summer seasons, and by 0.7236% in the fall. According to the Adaptive Neuro Fuzzy Inference System method, the projected dataset is consequently the most accurate and performs the best with the least accumulative mean absolute percentage error among the other techniques presented. As a result, the ANFIS data is significantly impacted by changes in the season of plug-in EVs’ per-hour charging use^41^.

In 2015, Khawaja et. al. proposed a study that the effect of many factors such as dry bulb, dew point temperatures, the hour of the weekday and day of each week, a flag indicator for weekends or holidays, average load from the day before, load from the same hour from the day before, and load from the comparable hour from the same weekday from the week before are just a few of the variables that the data has been taken into account in the forecasting model. The power load profile is taught and predicted using these criteria. As a result, forecasting results of the data collected from New England in the years 2004, 2005, and 2006 were produced using the suggested neural network-based methodologies, with monthly MAPE results not exceeding 8%^42^.

In 2024, El-azab et al. ^43^ discussed a study that discovered that developing a forecasting model has been the primary means of addressing the sharp rise and nonlinear behavior of the energy load and the price of power. ANN, LSTM, GRU, and ANFIS have been four prominent ways that have been suggested to increase forecasting accuracy and speed with a collection of mistakes root mean squared error “RMSE”, normalized root mean squared error “NRMSE”, mean absolute error “MAE”, and mean absolute percentage error “MAPE”. The ISO-NE electricity market is the source of the complete dataset for hourly energy load, hourly electricity price, and other factors. Therefore, they could run the forecasting model in two scenarios: the first one would forecast the energy load and the price of electricity separately, and the second would forecast the energy load and the price separately based on other factors like temperature parameters, day type, load during the same hour last week and the previous day, and price during the same hour last week and the previous day^43^.

Although it has been acknowledged that the second scenario’s forecasting model performs better and more efficiently than the first scenario’s in tracking actual values with the lowest MAPE, the nature of the electricity price in the deregulated market is still unstable and non-stationary. Where the datasets span the period of January 1, 2021, to December 31, 2021, or one year. The four seasons—winter, spring, summer, and autumn—have been represented by the dataset divisions. Following preprocessing and analysis of both the energy demand and electricity price datasets, the four featured algorithms in each of the two scenarios have been implemented. A collection of errors has been used to evaluate the four main algorithms. By comparing the findings from the two situations, it is discovered that ANFIS in the summer and winter provides the best predicting performance with the least amount of MAPE, and that the second scenario leads to a decrease in RMSE. The best forecasting performance is provided by ANN in the spring when MAPE is at its lowest and RMSE is reduced in the second scenario. However, the best forecasting performance with the lowest MAPE is provided by the autumn-season LSTM, while the second scenario has a lower RMSE^43^.

Additionally, the second scenario’s predicted outcomes from the machine learning and deep learning algorithms attempt to effectively track the actual values at the massive spikes that arise from the energy load’s non-linearity during specific seasons, which is reflected in the hourly electricity price. Because of the significant variations in the mean, standard deviations, minimum, and maximum for each season, there is a great deal of variability in both datasets. In addition, the second scenario requires more input parameters—like temperature, day type, hour-by-hour load, and price—than the first to produce anticipated outcomes that are more accurate and have less inaccuracy. The predicted results of the load and price that have emerged in minimizing the group of errors and tracking the actual values have been altered by the inclusion of external elements among the four seasons, particularly during the peaks of load and price. Furthermore, the load has been influenced by the cumulative effect of all the factors, such as the seasonal effects on weather temperatures and the weekday load profile, which is different on Mondays from the other weekdays. Furthermore, the hourly rate affects the load profile and is not fixed^43^.

According to the results in^44^, Random Forest performed the best at predicting power generation and wind speed, whereas the K Neighbors algorithm performed the worst and took the smallest amount of time to run^44^. Besides, S. M. Malakouti et al. in^45–48^ provided that the ensemble (light gradient boosting machine and Ada Boost) was used to forecast wind speed and solar farm production power of a supervisory control and data collection system to enhance the performance of the algorithms.

In addition to the ensemble approach, fourfold, fivefold, and tenfold cross-validation techniques were used to acquire the outcomes of machine learning algorithms. The algorithms’ outputs were contrasted with one another. The findings demonstrated that the ensemble method (light gradient boosting machine and Ada Boost) had a root-mean-square error of 11.78 with tenfold cross-validation in predicting the Supervisory control and data acquisition system’s three-month production power and 0.2080 with tenfold cross-validation in predicting the wind speed^45^.

Power plants that use fossil fuels will gradually be replaced by wind turbines as the main source of energy generation due to the scarcity of fossil fuels in many nations. These fossil fuel plants destroy the environment and increase the risk of disease in people and other living things. Investigations were conducted into wind turbines’ manufacturing potential. Thus, techniques like Multi-Layer Perceptron with Bayesian and XGBOOST^49^. CNN Long Short-Term Memory (CNN-LSTM), Ensemble (gradient boosting and xgboost), Gradient Boosting Regression Tree (GBDT), and Optimization (MLP + BO) have all been used. The Ensemble technique produced a mean square error (MSE) of 7.2 in 45 s, whereas the CNN-LSTM method produced an MSE of 6.8 in 450 s. Wind energy has the potential to be a dependable and sustainable energy source because it is easily accessible and affordable worldwide^49^.

Since most studies in the previous literature review have not been dependent on different factors as input parameters to the forecasting network such as temperature, day type (working day or special holiday), hour-by-hour load, hourly electricity costs and the seasonality effect in order to forecast the hourly load. Therefore, the enhancements that this work has contributed are outlined below:

- Building a forecasting network by utilizing large, high-quality datasets for two scenarios were provided in this manuscript to be utilized across the spring, summer, fall, and winter seasons. The two scenarios were:
- The machine learning and deep learning algorithms were chosen for training, testing, and predicting the load pattern were represented by the ANN, LSTM, GRU, and ANFIS.
- The suggested load forecasting models were analyzed using the four classes of errors root mean squared error “RMSE”, normalized root mean squared error “NRMSE”, mean absolute error “MAE”, and mean absolute percentage error “MAPE”, kurtosis coefficient, and coefficient of variation (CV) that were computed.
- The Nash–Sutcliffe Efficiency (NSE), and the determination factor (R^2^) established how accurate the simulated model was.

1. Forecasting the hourly load for one working day which was considered the last day in each season based on additional variables including dewpoints, dry bulbs, workdays, and electricity costs.
2. Forecasting the hourly load for one weekend day or special holiday in each season based on additional variables including dewpoints, dry bulbs, workdays, and electricity costs.

- The machine learning and deep learning algorithms were chosen for training, testing, and predicting the load pattern were represented by the ANN, LSTM, GRU, and ANFIS.
- The suggested load forecasting models were analyzed using the four classes of errors root mean squared error “RMSE”, normalized root mean squared error “NRMSE”, mean absolute error “MAE”, and mean absolute percentage error “MAPE”, kurtosis coefficient, and coefficient of variation (CV) that were computed.
- The Nash–Sutcliffe Efficiency (NSE), and the determination factor (R^2^) established how accurate the simulated model was.

The ISO (New England Independent System Operator) provided accurate data on the power market, which was where the information about hourly load, electricity prices, dewpoints, dry bulbs, workdays, weekends, and holidays originated in the four seasons. Four prominent algorithms—the Artificial Neural Network (ANN), the Long Short-Term Memory (LSTM), the Gated Recurrent Units (GRU), and the Adaptive Neural Fuzzy Inference System (ANFIS)—provided the results.

The paper contains eight sections. In brief, the machine and deep learning algorithms are displayed in Section "The recommended approaches". Section "Examining and preparing the dataset for processing" displays the examination and preparing the dataset for processing. Section "Seasonal short-term load forecasting procedure" describes the seasonal short-term load forecasting procedure. Section "Seasonal energy load consumption and tuning parameters of the suggested algorithms under study" proposes seasonal energy load consumption and tuning parameters of the suggested algorithms under study. Prediction results and discussion are discussed in section "Results of short-term forecasting model and discussion" discussion and the advantages of the proposed study are summarized in section "Discussion and the benefits of the provided study". Section 8 points to the conclusion of this study.

## The recommended approaches

This section contains representations of the suggested algorithms. On the other hand, it would be better to introduce the model’s framework for short-term forecasting of the profile in four seasons. The section explains the suggested algorithms: artificial neural fuzzy inference systems, gated recurrent units, and long short-term memory.

### Long short-term memory (Lstm) topology

**Fig. 1:**
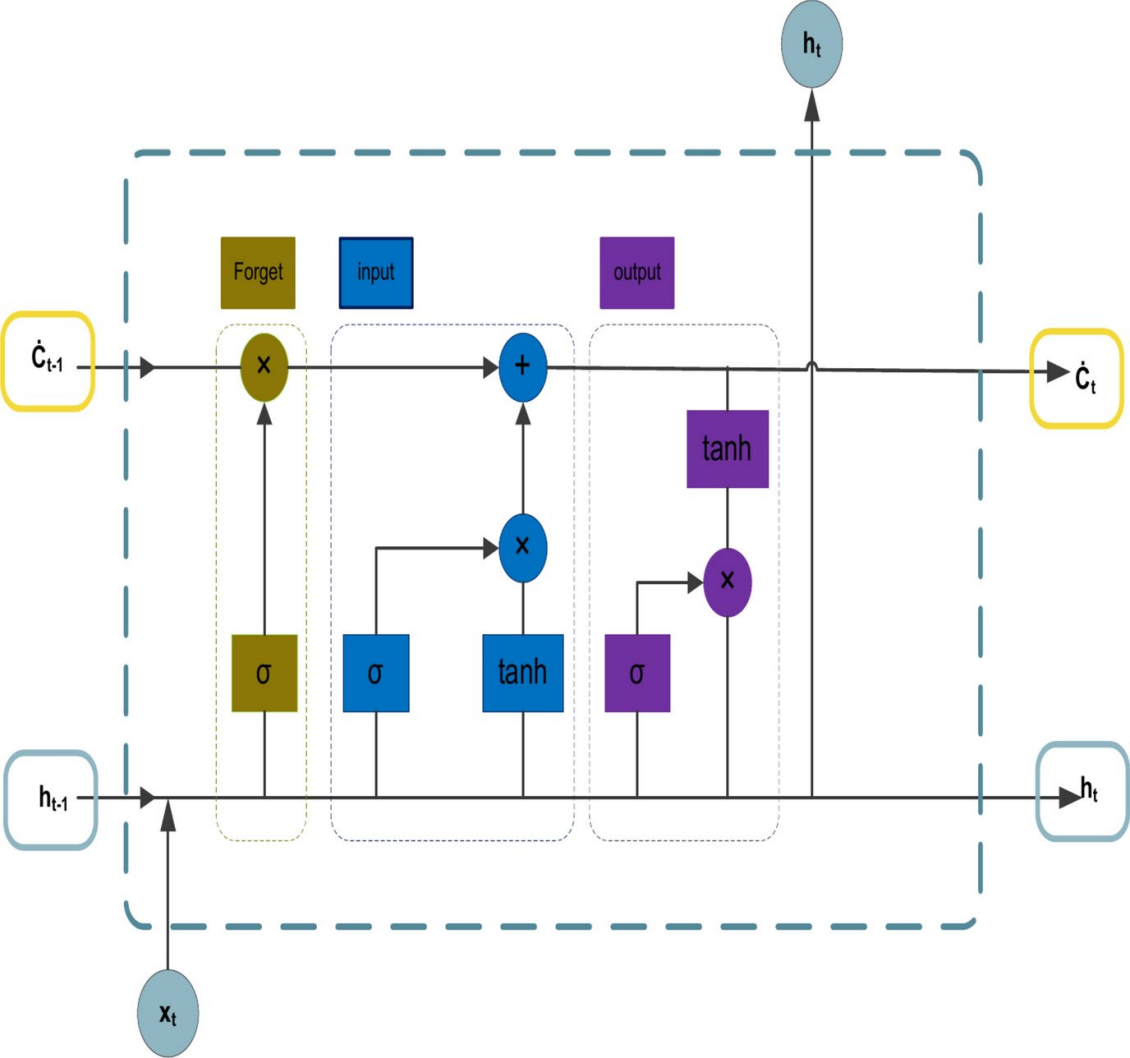
A structure model of the LSTM network^53^.

An ANN type called an RNN manages prerequisites between nodes of information. The RNN model incorporates the notion of the concealed state. After alterations, the data’s eigenvalues can be extracted from the hidden state. For short-term dependency problems, RNNs work well. However, the long-term dependence issue is beyond the model’s capabilities. The gradient disappearance dilemma was the motivation behind the creation of the LSTM network. A gate control method is introduced by the LSTM network. The forgetting gate determines the data that is the subject of cell state loss, the information to be output is determined by the output gate, and the cell state to store new information is determined by the input gate^50,51^. Figure 1 depicts LSTM’s topology. The cell’s state at t is introduced by $\check{C}_{t}$ and the forget gate is indicated by $F_{t}$. The input gate is indicated by $i_{t}$. $\overline{{\mathop {\text{O}}\limits^{.} }}_{t}$ stands for the output gate. Its formula in mathematics is^52^:

1 $$F_{t} = \sigma {(}\omega_{F} \cdot \left[ {h_{t - 1} ,\chi_{t} } \right] + \underset{\raise0.3em\hbox{$\smash{\scriptscriptstyle\cdot}$}}{\text {b}} )$$

2 $$i_{t} .{ } = \sigma \left( {\omega_{{\underset{\raise0.3em\hbox{$\smash{\scriptscriptstyle\cdot}$}}{\text{i}}}} \cdot \left[ {h_{t - 1} ,\chi_{t} } \right] + \underset{\raise0.3em\hbox{$\smash{\scriptscriptstyle\cdot}$}}{\text{b}}_{{\underset{\raise0.3em\hbox{$\smash{\scriptscriptstyle\cdot}$}}{\text{i}}}} } \right)$$

3 $${\hat{\text{C}}}_{t} = tanh\left( {\omega_{{{\dot{\text{C}}}}} \cdot \left[ {h_{t - 1} ,\chi_{t} } \right] + {\dot{\text{b}}}_{{{\dot{\text{C}}}}} } \right)$$

4 $${\dot{\text{C}}}_{t} = F_{t} * {\dot{\text{C}}}_{t - 1} + i_{t} * {\dot{\text{C}}}_{t}$$

5 $$\underset{\raise0.3em\hbox{$\smash{\scriptscriptstyle\cdot}$}}{\text{O}}_{t} = \sigma \left( {\omega_{{\underset{\raise0.3em\hbox{$\smash{\scriptscriptstyle\cdot}$}}{\text{O}}}} \cdot \left[ {h_{t - 1} ,{\dot{\text{X}}}_{t} } \right] + \underset{\raise0.3em\hbox{$\smash{\scriptscriptstyle\cdot}$}}{\text{b}}_{{\underset{\raise0.3em\hbox{$\smash{\scriptscriptstyle\cdot}$}}{\text{O}}}} } \right)$$

6 $$h_{t} = \underset{\raise0.3em\hbox{$\smash{\scriptscriptstyle\cdot}$}}{\text{O}}_{t} * \tanh {\dot{\text{C}}}_{t}$$

### Gated recurrent units (GRU) topology

**Fig. 2:**
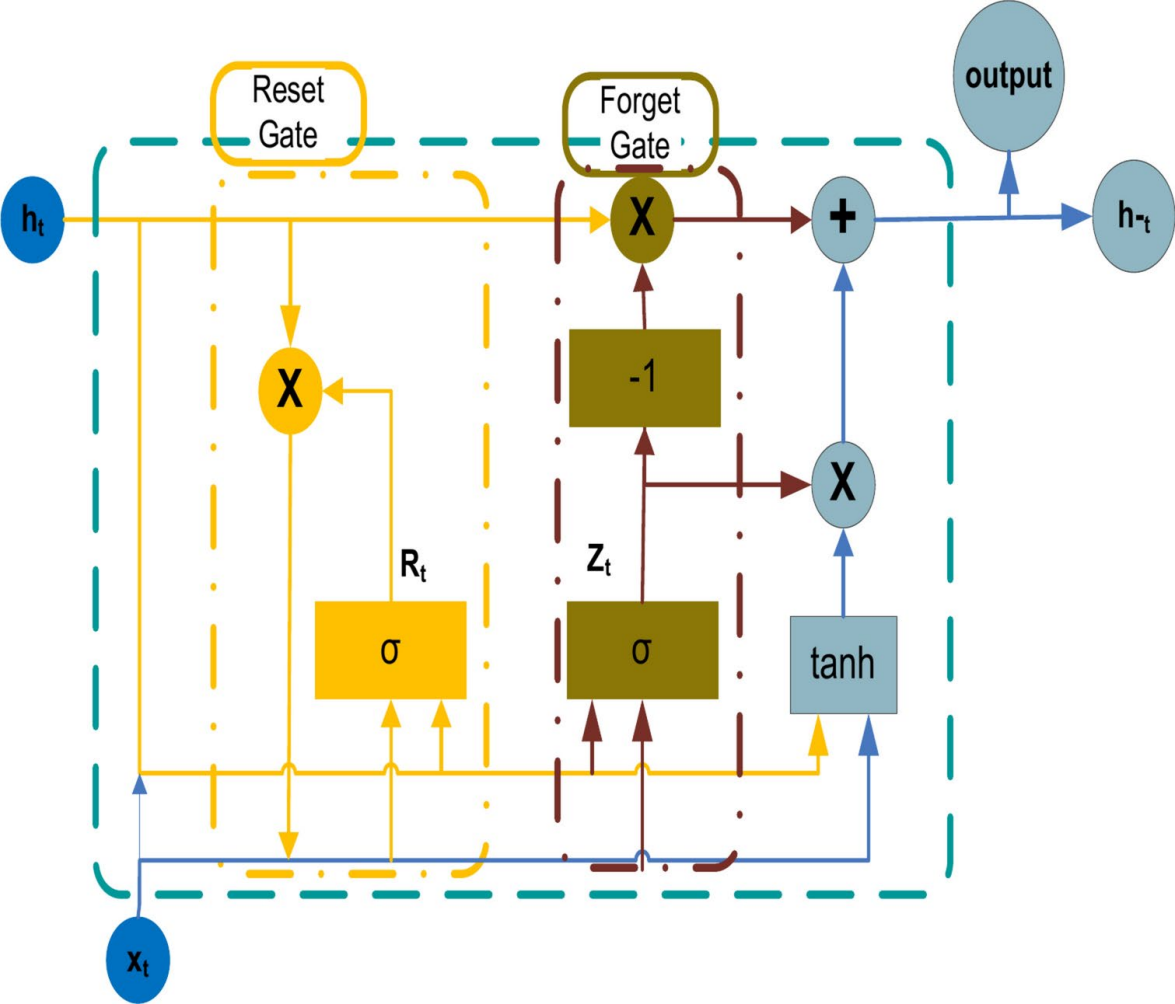
A structure of the GRU^57^.

A gating technique for recurrent neural networks called gated recurrent units (GRUs) was developed in 2014^55^. The GRU has fewer settings than an outcome gate, however, it is comparable to an LSTM (long short-term memory) with a forget gate^56^. It has been shown that GRUs function superior on records that are smaller and less frequent^57^. GRU, which represents progress on the concealed layer of the standard RNN, is shown graphically and architecturally in Figure 2. An update gate, a reset gate, and a temporary output are the three gates that comprise a GRU. The following are the related symbols^57^:

At time $t$, the framework input signal is represented by the parameter $x_{t}$.
The values of the vectors represented by variables $h_{t}$ and $h_{t}^{ - }$ at moment t are the transient outcome and the concealed layer output, respectively.
The gate vectors $Z_{t}$ and $R_{t}$, represent the variables’ outcome of the modified and reset gates at t, respectively.
(X) and tanh(X) indicate the sigmoid and tanh activation functions, respectively.

### Adaptive neural fuzzy inference system (ANFIS) topology

**Fig. 3:**
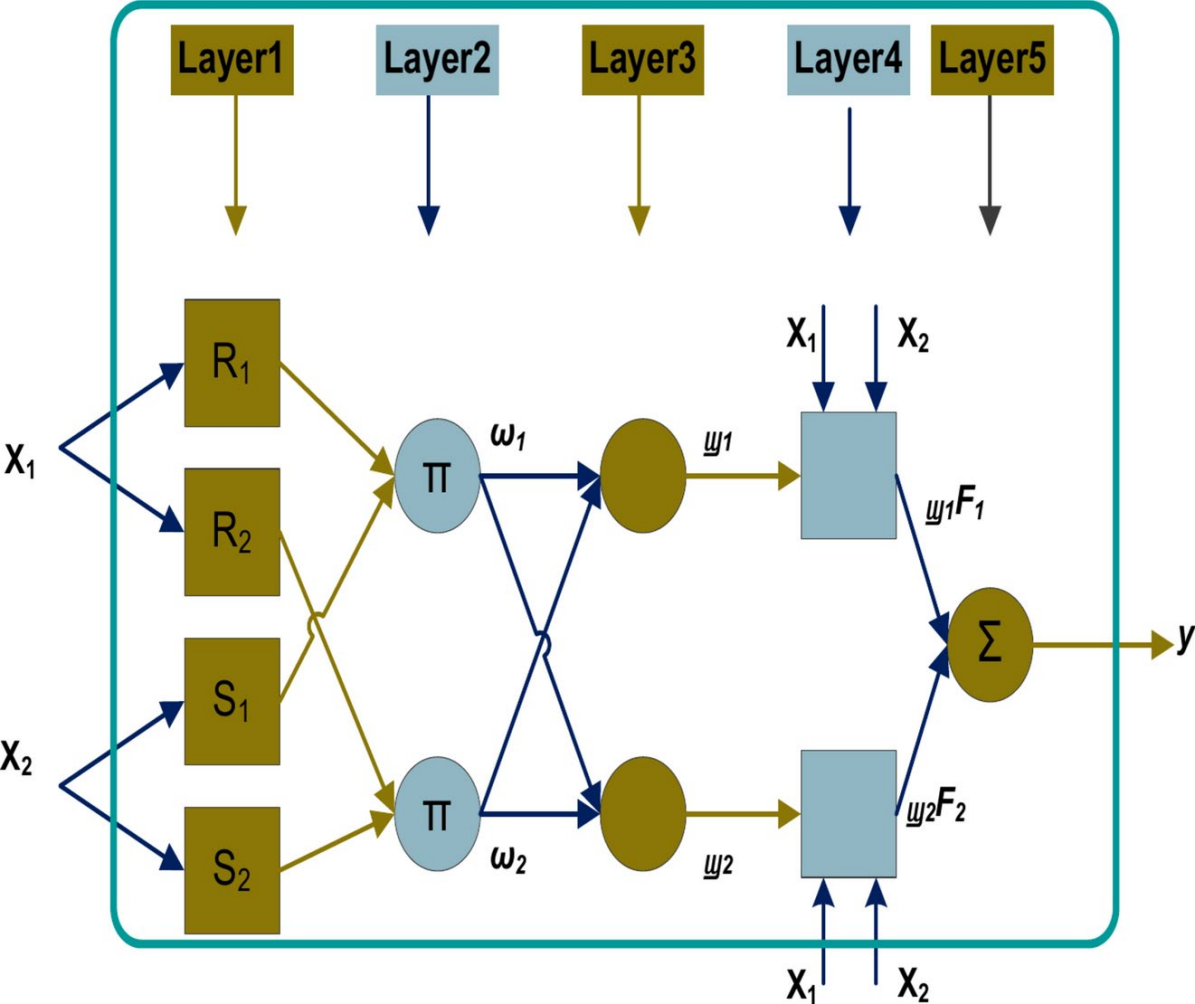
The fundamental architecture of ANFIS for a single output with two rules and two inputs^58^.

Figure 3 depicts the structure of the ANFIS. The ANFIS approach forms a fuzzy inference system (FIS) from a given the term "input” and the term “output” sets of data. The membership function parameters of the FIS are then configured employing a least squares-type approach or backpropagation technique independently. As a result, fuzzy structures can gain knowledge from the data that they simulate^58,59^. The FIS Design is a network structure that resembles a neural network in that it correlates inputs to outputs via output membership functions and related parameters after mapping inputs through input membership functions and corresponding factors. To identify the ideal membership function distribution using hybrid learning, an ANFIS can assist us in identifying the mapping relationship between the input data and outcome^57^.

The method employed by ANFIS can be implemented without just depending on the skills of expertise required for a fuzzy logic methodology. The ANFIS technique has the benefits of possessing both quantitative and language competence. ANFIS also makes use of the ANN’s capacity to categorize information and determine correlations. Compared to the ANN, the ANFIS structure is easier for users to understand and is less prone to cause memorization errors. Consequently, the ANFIS offers several benefits, including nonlinearity, rapid learning and training, and adaptability^58^.

In essence, the ANFIS technique is a fuzzy logic strategy based on regulations, in which the regulations are developed during the algorithm’s training phase. The method is based on data-driven learning. The samples that are trained are used by ANFIS to construct the fuzzy inference system (FIS) function with its membership specifications. The two systems of fuzzy inference that are most commonly used are Mamdani and Sugeno. The key difference between the Sugeno and Mamdani procedures is that the membership outcome functions for the Sugeno approach can be fixed in nature or regular. However, the membership outcome functions of the Mamdani approach might be Gaussian, triangular, etc. This study employed the Sugeno-type fuzzy inference system since it computes more quickly than the Mamdani type. The Mamdani rely largely on knowledge. On the other hand, the Sugeno category was created using real data^58–61^.

**Fig. 4:**
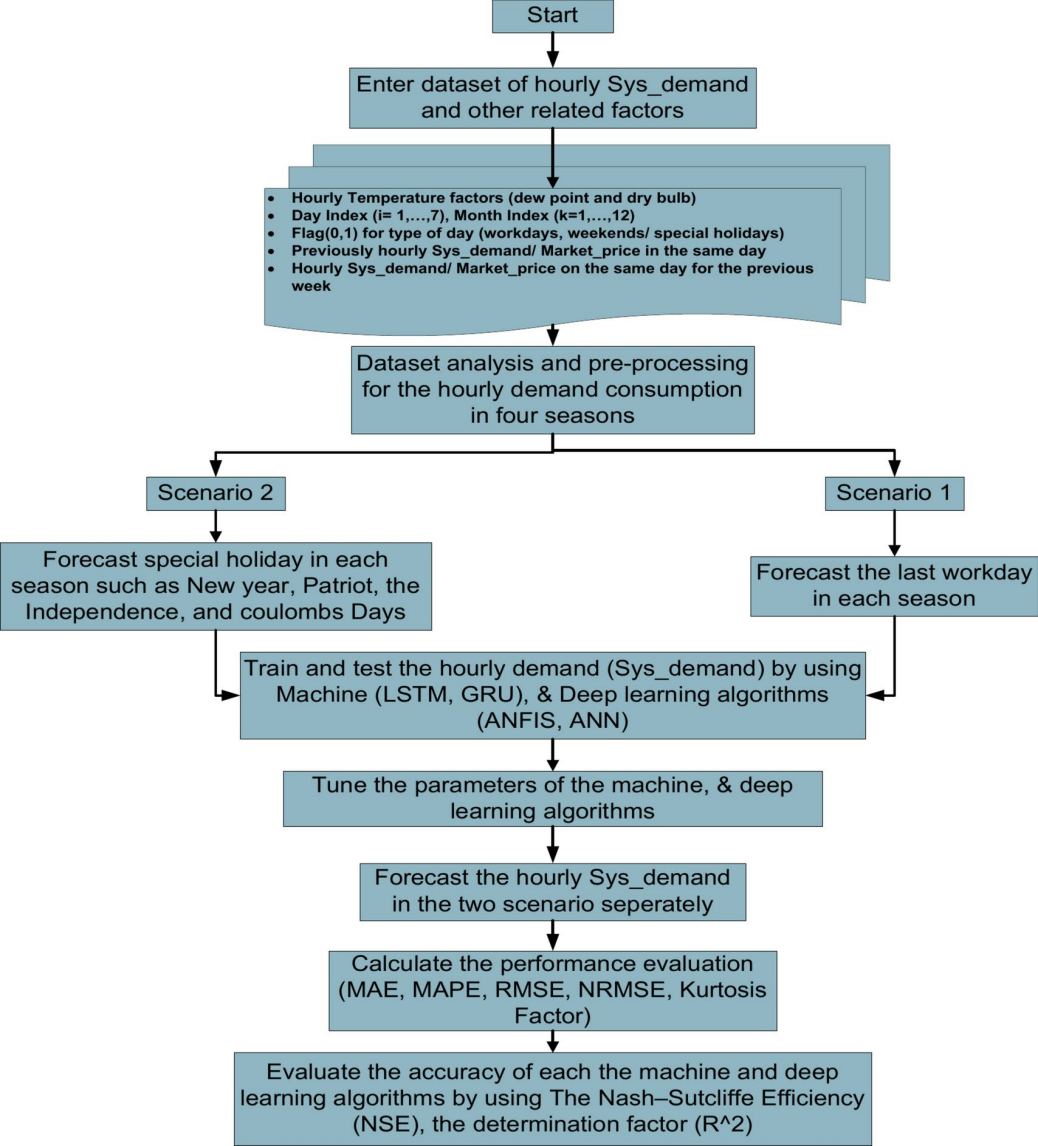
Predicting the hourly energy load framework using the two scenarios’ performance calculations and the four suggested methodologies.

To comprehend the ANFIS framework, we made the assumption that there are, in fact, two inputs: x and y. A double set of fuzzy if-then rules for a first-order Sugeno fuzzy framework can be mentioned in the following way in rules 1 and 2:

7 $${\text{Rule }}1:{\text{ if x}}_{1} {\text{ is }}R_{1} {\text{and }}x_{2} {\text{ is }}S_{1} {\text{then }}\left( {F_{{1{ }}} = { }a_{1} x_{1} + { }b_{1} x_{2} + { }c_{1} } \right)$$

8 $${\text{Rule }}2:{\text{ if }}x_{2} { }is{ }R_{2} a{\text{nd }}x_{2} { }is{ }S_{2} {\text{ then }}\left( {F_{{2{ }}} = { }a_{2} x_{1} + { }b_{2} x_{2} + { }c_{2} { }} \right)$$

where $a_{{\text{i}}}$, $b_{{\text{i}}}$ and $c_{{\text{i}}}$ are the arrangement of elements that are determined throughout the model-learning process, $R_{{\text{i}}}$ and $S_{{\text{i}}}$ are the fuzzy sets, and $F_{{\text{i }}}$ is the outcome. The ANFIS framework that was used to carry out the two rules is shown in Fig. 4. The two inputs that are provided to the fuzzy system, ${\text{x}}_{1}$ and ${\text{x}}_{2}$, will therefore be used to build layer 1’s fuzzy function of membership^58^.

1. Equations (9) through (12) illustrate the four-level fuzzification of the two inputs, ${\text{x}}_{1}$ and ${\text{x}}_{2}$, to get the result Y. **The first layer of the fuzzy system** will receive the two inputs. The membership functions ${\upmu }_{{R_{i} }} \left( {{\text{x}}_{i} } \right)$ and ${\upmu }_{{S_{{\text{i}}} }}$(${\text{x}}_{i}$) are employed when the return of layer 1 is $O_{i,1}$,. The membership criteria include Gaussian in nature trapezoidal, and triangle-shaped forms^58^.
  $$O_{i,1} = { }\mu_{{R_{i} }} \left( {x_{i} } \right),{\text{ for }}i{ } = 1,{ }2$$
  9 $$O_{i,1} = { }\mu_{{S_{{\text{i}}} }} \left( {x_{i} } \right),{\text{ for }}i{ } = 3,{ }4$$
2. **The second layer of the fuzzy system**, when $i$ = 1, 2, the fuzzy rules are represented by Eq. (4).
  10 $$O_{i,2} = { }\omega_{i} = \mu_{{R_{i} }} \left( {\chi_{i} } \right) \mu_{{S_{i} }} \left( {\chi_{i} } \right),{\text{ for }}i{ } = 1,{ }2$$
3. The gate vectors $Z_{t}$ and $R_{t}$, represent the variables’ outcome of the modified and reset gates at t, respectively.

1. **The third layer of the fuzzy system**, Eq. (11) illustrates the normalization of this layer’s output.
  11 $$O_{i,3} = { }w_{i} { } = \frac{{ \omega_{1} }}{{ \omega_{1} + \omega_{2} }},{\text{ for}} i{ } = 1,{ }2$$
2. **The fourth layer of the fuzzy system,** the output of the membership function is shown in Eq. (12).
  12 $$O_{i,4} = { }w_{i} F_{i} { } = w_{i} \left( {a_{i} x_{1} + b_{i} x_{1} + c_{i} } \right) ,{\text{ for }}i{ } = 1,{ }2$$
3. **The fifth layer of the fuzzy system**, and for summary, all incoming signals will go through the activation function, which will cause them to become fuzzy, and the fuzzy rules, which will cause them to become less fuzzy. Ultimately, the outcome is ready for assessment, as indicated by Eq. (13).
  13 $$O_{i,5} = { }\mathop \sum \limits_{i = 1}^{2} w_{i} F_{i} { } = \frac{{\mathop \sum \nolimits_{i = 1}^{2} \omega_{i} f_{i} }}{{ \omega_{1} + \omega_{2} }},{\text{ for }}i{ } = 1,{ }2$$

## Examining and preparing the dataset for processing

Throughout the data preparation phase, a suitable structure is produced for the analytical task. Because of this, data preparation is necessary in order to modify the data and effectively assess the results.

### Dataset normalization and de-normalization procedures

When evaluations are made using scales that differ, like in the case of forecasting inputs. Ignoring this stage, especially when working with gradient descent approaches, causes the convergence rate to decrease to a minimum and distorts the usefulness of the outcomes by preventing them from gathering data. The normalizing technique used in almost all the publications is called min–max scaling, and it turns each value in the dataset into an interval between zero and one^21^. Owing to the significant variations in the target data, the normalization process helps to enhance the framework’s performance during learning, accelerating the training stage. The formulation of the min–max normalization is given by Eq. (14)^62^.

14 $$y = \frac{z - \min \left( z \right)}{{\max \left( z \right) - \min \left( z \right)}}$$

where the actual value is $z$, max ($z$), and min ($z$) are the maximum and the minimum values of $z$, and the normalized value is $y$^62^.

After being achieved, the predicted results of the framework ought to automatically be returned to their initial values for improved readability. This process is also known as denormalization or anti-normalization^63^. The normalization and the anti-normalization processes are carried out on the hourly load and the corresponding hourly price in order to easily deal with the pattern label each month.

#### Handling missing values and the outlier procedure

Throughout the outlier stage, a few ways are used to remove disruption and missing information that cause fatal errors in model-based forecasting. This process is advised when the zero data is eliminated after being transformed to null^63^.

### Datasets clustering procedure

When pre-processing the datasets of the energy load, clustering may be employed to separate the database into seasons, patterns of the weather, or type of day such as a flag 0 or 1 to differentiate between the working day, day off (weekend, or special holiday)^63^. The clustering method is carried out on the hourly energy load and the related hourly price to clarify the pattern label each month.

#### Dataset smoothing procedure

Dataset assembly allows for the removal or reduction of fluctuations and various types of disruption. This is known as data softening^64^. In this paper, the hourly energy load dataset and the corresponding hourly price are smoothed using the moving average method.

## Seasonal short-term load forecasting procedure

Predicting the electric energy load under two scenarios is the goal of this study. The first scenario involves Predicting 24 h for one working day, the final day of each season, taking into account several variables including dewpoints, dry bulbs, workdays, and the corresponding electricity costs of consumption. In the second scenario, predicting 24-h for one weekend day or special holiday every season, taking into account other variables including dewpoints, dry bulb conditions, off days, and the corresponding electricity costs of the consumption in the same season.

A forecasting load model framework based on the suggested machine learning and deep learning algorithms is shown in Fig. 4 for the two scenarios. These algorithms are described as follows:

1. As the three phases in the analysis and preprocessing stage, the dataset is run through outlier, normalization, de-normalization, clustering, and smoothing in the two scenarios.
2. Each suggested algorithm is prepared for application on the dataset in the input matrix for the load/price for the two forecasting model scenarios following the pre-processing of the dataset.
3. There are separate datasets for training and testing for the hourly demand in each season.
4. Using the optimizing solver and loss function to fine-tune each algorithm’s parameters throughout the training phase.
5. Use individual machine and deep learning algorithm simulations to forecast the outcome matrix for the hourly load in the two scenarios.
6. Use the measured performance root mean squared error “RMSE”, normalized root mean squared error “NRMSE”, mean absolute error “MAE”, and mean absolute percentage error “MAPE”, kurtosis coefficient, and coefficient of variation (CV) to assess how well each method performed in obtaining results for short-term load forecasting in the two scenarios.
7. Finally, an accurate simulation of the forecasting model is assessed using the determination coefficient R^2^.

It is crucial to decide on the best possible optimizer and loss function for the suggested algorithms to achieve optimal training results. The optimizer is a mathematical strategy that reduces losses in the suggested algorithm by adjusting its weights, bias, and learning rate, among other qualities. Consequently, by fine-altering the settings for each suggested algorithm and choosing the proper optimizer and loss function, the discrepancy between the tested and predicted data is decreased.

Software for engineering uses many efficient optimizing solvers, including the Levenberg–Marquardt algorithm (LMA)^65^, Adaptive Gradient (AdaGrad)^66^, Adaptive Delta (Adadelta)^67^, Adaptive Moment Estimation (Adam)^66^, and Nesterov Accelerated Gradient (NAG)^68^.

In this study, we select the most efficient and the most optimizing solver in the following manners:

1. Adaptive Moment Estimation (Adam) is selected for ANFIS, GRU, and LSTM algorithms.
2. The Levenberg–Marquardt algorithm (LMA) is selected for the ANN algorithm.

Additionally, the following is the helpful loss function corresponding to the best-optimizing solver:

1. Mean Squared Error (MSE) is selected for ANFIS, GRU, and LSTM algorithms.
2. Sum Squared Error (SSE) is selected for the ANN algorithm.

Where Eqs. (14) and (15) can be used to compute the Mean Squared Error (MSE) and Sum Squared Error (SSE). Where the number of samples is indicated as “N”, and the forecasted and actual values of the energy load consumption (Sys_demand) are indicated as $z_{{\text{t}}} ,{\text{ and }}y_{{\text{t}}}$, respectively^68^.

14 $${\text{SSE}} = { }\mathop \sum \limits_{{{\text{t}} = 1}}^{{\text{N}}} \left( {{ }z_{{\text{t}}} - y_{{\text{t}}} } \right)$$

15 $${\text{MSE}} = { }\frac{{\mathop \sum \nolimits_{{{\text{t}} = 1}}^{{\text{N}}} \left( {{ }z_{{\text{t}}} - y_{{\text{t}}} } \right)}}{{\text{N}}}$$

## Seasonal energy load consumption and tuning parameters of the suggested algorithms under study

The dataset for the energy consumption load is shown in this section. In order to produce better-predicting results with less error, the setting parameters of each algorithm are adjusted to make it adequate to function with the dataset.

### Compiling the dataset procedure

The New England power market, ISO-NE, is the source of the statistics. The Independent System Operator New England, also known as ISO-NE, is the organization in charge of producing, handling, and distributing electricity to end users or customers. ISO-NE offers a lot of data, including load, dew points, dry bulb, pricing, supply of energy, and generation of energy. For a year, from January 1, 2021, to December 31, 2021, this study used hourly load and pricing data each day from Independent System Operator New England (ISO-NE) (https://www.iso-ne.com, accessed on: October 7, 2023). The data’s objective is to predict the energy load consumption of power in the aforementioned two scenarios. “Sys_demand” and “Market_Price” columns in our target records are indicated^70^.

**Fig. 5:**
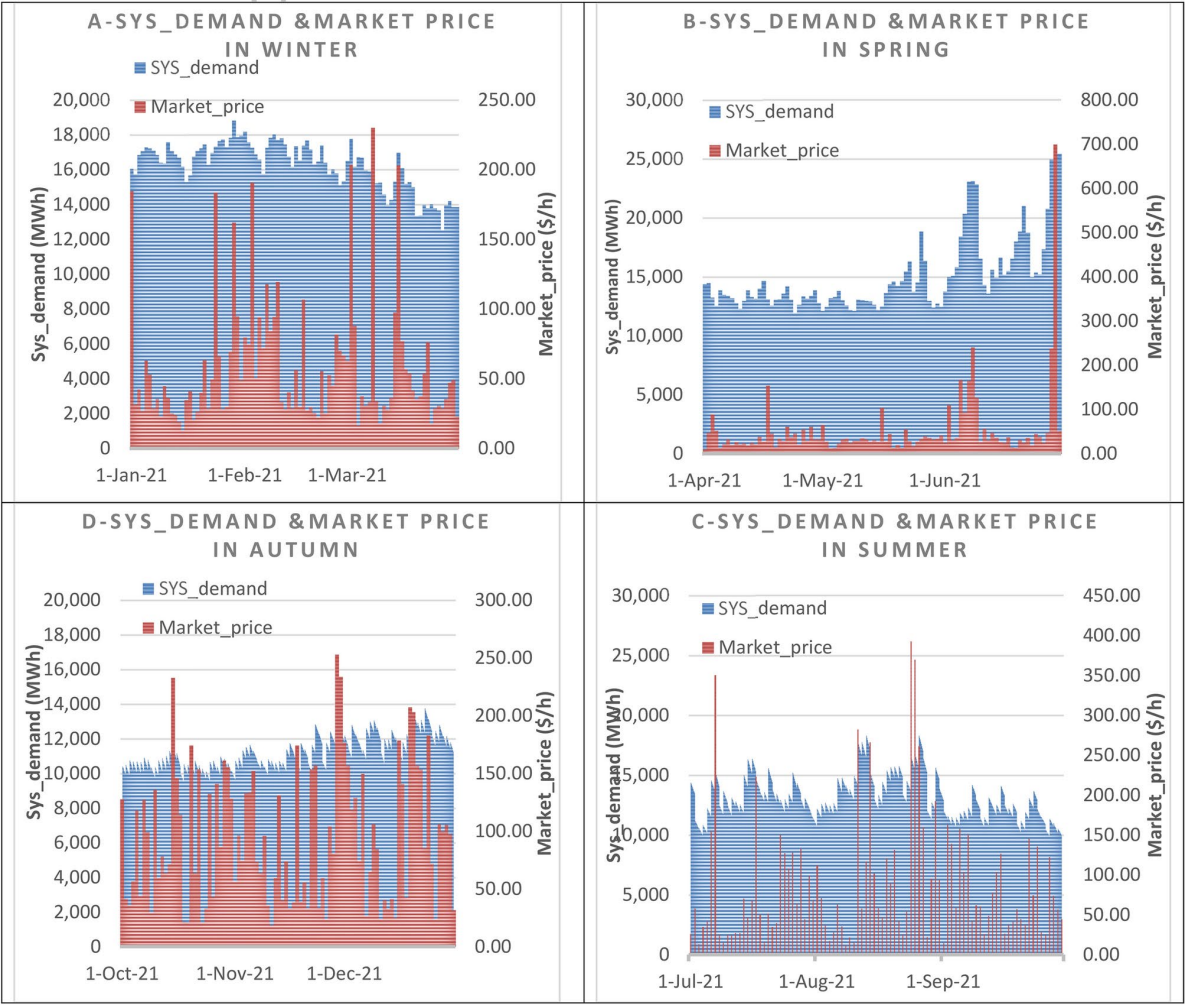
The Sys_demand (MWh) hourly dataset for the four seasons, linked with the associated hourly Market_price ($/h)^70^.

Since the dataset was collected for twelve months, there are 8760 records. Consequently, the dataset is divided into two sets: the one used for the training set and the one used for the testing set. Since the forecasting framework will develop the datasets used to train the suggested algorithm. The dataset for each of the four seasons which is the hourly energy load consumption (Sys_demand) in MWh is displayed in Fig. 5. In addition, the corresponding dataset of the Market_price is considered as one of the inputs that affects forecasting the hourly energy load consumption (Sys_demand) is also shown in Fig. 5. Before beginning the analysis and pre-processing procedures. Two scenarios exist:

1. In the 1st scenario, the hourly energy load consumption (Sys_demand) is forecasted for the workdays, based on some elements such as temperature, the power price in the electricity market, and the previous workdays (demand, and market price) profile in the same season.
2. In the 2nd scenario, the hourly energy load consumption (Sys_demand) is forecasting for the weekend days or even special holidays, based on some elements such as temperature, the power price in the electricity market, the previous weekend (demand, and market price) profile in the same season.

The input factors and the outcome for the two scenarios are displayed in Table 1. The temperature, expressed in Celsius degrees, the Market_price, and the load are based on the same-hour price and load from the previous day, the same-hour price and load from the previous week, and the preceding 24-h average price and load. These correspond to three distinct calendar index flags, namely the day numbering of the week, the month, and type of the day where the day number flag is i = 1, 2, …, 7 for the seven days of the week (Monday, Tuesday, …, Sunday), the month indication is k = 1, 2, …, 12 for the twelve months (January, February, March, …, December), and type of the day flag is f = 0, 1 for the weekend or holidays, and the working days.

**Table 1:** The input parameters and the outcome.

Input/Output	Parameters	Unit
Input	Temperature in each hour	°C
Input	Demand/Price of the hour, a day, a week before	MWh/$/h
Input	Month Index	K = 1, 2, 3, …, 12
Input	Day Index	i = 1, 2, 3, …, 7
Input	Day Type (weekend/holiday, workday)	F = 0, 1
output	Hourly Sys_demand	MWh

We suppose that the temperature records for a given year are divided over the four seasons. The range of temperatures within winter, spring, summer, and fall is therefore considered a seasonal factor in this study. For example, the average wintertime temperature ranges from − 1/− 11 to 5/− 5 °C for the highest and lowest points. For the spring, both the maximum and minimum mean temperatures have been between 12/1 and 24/12 °C. The mean variation in temperature between the highest and lowest points was 27/16 and 21/10 °C. During autumn, the average maximum and minimum temperatures have fluctuated between 14/4 and 2/− 7 °C^71^.

.

**Fig. 6:**
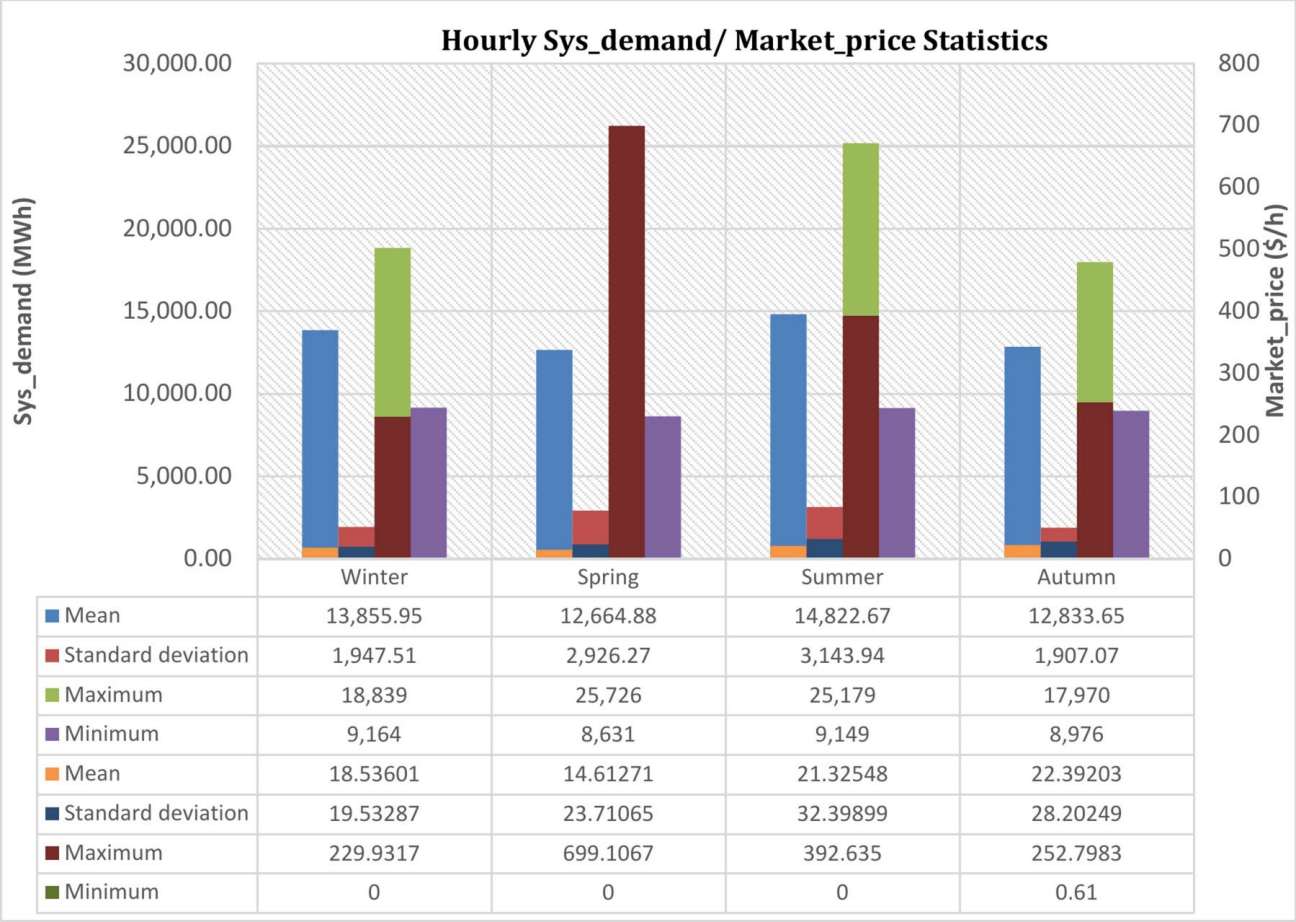
Data from statistics on hourly Sys_demand and the corresponding Market_price throughout the four seasons.

Figure 6 offers detailed information on the electricity pricing and energy load statistics utilized in this analysis, which were collected from the deregulated market (ISO-NE). Because of the significant variations in the mean, standard deviation, maximum, and minimum of the hourly energy demand consumption and the Market_price records, Fig. 6 shows the average, standard deviation, maxima, and minima of the datasets in the four seasons respectively. This shows large magnitude variations and swings in the hourly energy demand and Market_price records. Because of this, irregular samples cannot be simulated by traditional statistical models like ARMA and ARIMA. Given the non-linearity and fluctuation characteristics of the forecasting model, machine and deep learning approaches can be used to train and test the datasets.

### Tuning the specifications of the selected algorithms

1. The dataset is split in both the two scenarios into 70% allocated for the training set and 30% allocated for the test set.
2. Four seasons, lasting three months apiece, are represented in the data being modeled. Where:
  January, February, and March are considered the wintertime months.
  April, May, and June are considered springtime.
  During July, August, and September are the summertime months.
  October, November, and December are considered the fall months.
3. Workdays/ weekends and special holidays according to calendar 2021. The working days are from Monday to Friday, and the weekend days in this study are considered as a special holiday that may be followed by weekend days in the calendar.
  For workdays, the last day in each season is forecasted such as 31 March in winter, 30 June in spring, 30 September in summer, and 31 December in autumn.
  For weekends which are considered special holidays in this study, these days are forecasted in each season such as 1 January in winter which is New Year’s Day, 19 April in spring, which is Patriot’s Day, 5 July in summer which is the Independence Day, and 11 October in autumn which is the Coulombs’ Day.
4. The four suggested approaches—ANN, LSTM, GRU, and ANFIS—simulate the acquired findings.

#### ANN Algorithm tuning specifications

For the ANN algorithm, the Sum Squared Error (SSE) measures performance statistics. The internal optimizing solver Levenberg-Marquardt algorithm (LMA) trains the dataset to find the least SSE as the most efficient loss estimator. The following is how the other settings are tuned:

- 10 neurons are started in 3 layers. Where the three layers are composed of input, hidden, and outcome layers.
- [6, 1] is the value of the input weight "W_i_".
- [2, 2] is the value of the layer weight "W_l_".
- [6, 1] is the value of the bias "b".

#### LSTM and GRU algorithms tunning specifications

For both LSTM and GRU algorithms, Mean Squared Error (MSE) measures performance statistics. The internal optimizing solver Adaptive Moment Estimation (ADAM) trains the dataset to find the least MSE as the most efficient loss estimator. The following is how the other settings are tuned:

- In the instance of the two scenarios, there are six inputs, resulting in one output.
- The maximum epoch value is 500.
- The number of iterations is 50, and the number of hidden layers is 10 hidden units.
- The gradient threshold has a value of 1.
- The “piecewise” initial learn rate and its schedule are 0.005.
- The values of factor and learning rate drop period are 0.2 and 125, respectively.

#### ANFIS algorithm tuning specifications

For the ANFIS algorithm, Mean Squared Error (MSE) measures performance statistics. The internal optimizing solver Adaptive Moment Estimation (ADAM) trains the dataset to find the least MSE as the most efficient loss estimator. The following is how the other settings are tuned:

- There are 128 nodes in all.
- There are 100 nonlinear parameters.
- There are 247 training data pair pairings.
- There are ten fuzzy rules.
- The maximum epochs have a value of 100.
- There is zero mistake aim.
- The starting step size is 0.1. 0.9, and 1.1, respectively, for the reduction and increase rates.

## Results of short-term forecasting model and discussion

This section assesses the performance of the four suggested algorithms—ANN, LSTM, GRU, and ANFIS—after preprocessing, training, and testing the dataset of the hourly load consumption (Sys_demand). Next, utilizing a group of errors, the outcomes from the two scenarios are evaluated. The MATLAB 2021a edition with 16.00 GB RAM and an Intel® Core^TM^ i7-9750H CPU running at 2.60 GHz is used to simulate the results.

### Emulation performance of the outcomes

The load in this instance may be estimated using the mean and standard deviation of the variable set as follows in Equations (16) through (17)^72^:

16 $$\overline{x} = \frac{1}{n} \mathop \sum \limits_{i = 1}^{n} x_{i}$$

17 $$\sigma = \frac{1}{n} \mathop \sum \limits_{i = 1}^{n} (x_{i} - \overline{x})$$

where σ is the standard deviation of load $x$, $x_{i}$ is the load at time i, n is the load data length or forecasting horizon, and $\overline{x}$ is the load mean. To calculate the coefficient of variation $CV$ by the following Eq. (18)^72^:

18 $$CV = \frac{\sigma }{{\overline{x}}}$$

To assess the efficacy of the four suggested techniques, performance is evaluated using a set of errors, including mean absolute error (MAE), mean absolute square error (RMSE), normalized root mean square error (NRMSE), and mean absolute percentage error (MAPE)^43^.

The calculations of RMSE, NRMSE, MAE, and MAPE can be performed using Equations (19) through (22)^43,73,74^.

19 $$RMSE = \sqrt {\frac{1}{n} \mathop \sum \limits_{i = 1}^{n} \left( {z_{i} - y_{i} } \right)^{2} }$$

20 $$NRMSE = \frac{{\sqrt {\frac{1}{n} \mathop \sum \nolimits_{i = 1}^{n} \left( {z_{i} - y_{i} } \right)^{2} } }}{{{\overline{\text{z}}}}}$$

21 $${\text{MAE}} = \frac{1}{{\text{n}}}{ }\mathop \sum \limits_{{{\text{i}} = 1}}^{{\text{n}}} { }\left| {z_{{\text{i}}} - {\text{ y}}_{{\text{i}}} } \right|$$

22 $${\text{MAPE}} = \frac{1}{{\text{n}}}{ }[\mathop \sum \limits_{{{\text{i}} = 1}}^{n} { }|\frac{{z_{{\text{i}}} - { }y_{{\text{i}}} }}{{y_{{\text{i}}} }}|{ }]{ *}100{\text{\% }}$$

where "n" is the number of samples, and "${\text{z}}_{{\text{i}}}$" and "${\text{y}}_{{\text{i}}}$" are the predicted and actual values, respectively, for a given time "i". On the other hand, ${\overline{\text{z}}}$ represents the time series’ average values for sample “n”.

The kurtosis coefficient is a statistical measure that describes the shape of a probability distribution. Specifically, it quantifies the “tailedness” of the distribution, or how much data is concentrated in the tails compared to the center^72,73^.

Where the types of Kurtosis:

- Leptokurtic: Distributions with high kurtosis. They have heavier tails and a sharper peak than a normal distribution. This means there’s a higher probability of extreme values (outliers).
- Mesokurtic: Distributions with a kurtosis similar to a normal distribution. They have moderate peaks and moderate tails.
- Platykurtic: Distributions with low kurtosis. They have lighter tails and a flatter peak than a normal distribution. This means there’s a lower probability of extreme values.

The range of values for the kurtosis coefficient is:

- For positive kurtosis (kurtosis greater than 3): 1 to infinity
- For negative kurtosis (kurtosis less than 3): − 2 to infinity

Equation (23) describes the Kurtosis that provides details about the shape. A sharper peak surrounding the distribution’s mode is indicated by positive kurtosis. In addition, a distribution with more extreme values than a normal distribution is indicated by a larger kurtosis. As a result, a forecasting model with a high kurtosis shows more outliers that deviate from the error distribution mean^73^.

23 $$Kurtosis = \frac{n}{{\left( {n - 1} \right)\left( {n - 2} \right)\left( {n - 3} \right)}} \mathop \sum \limits_{i = 1}^{n} \left( {\frac{{e_{i} - \overline{{e_{i} }} }}{{\sigma_{{e_{i} }} }}} \right)^{4} - \frac{{3\left( {n - 1} \right)^{2} }}{{\left( {n - 1} \right)\left( {n - 3} \right)}}$$

where "n" is the number of samples, "${\text{e}}_{{\text{i}}}$" is the error between predicted and actual values, and "$\overline{{e_{i} }}$", and “$\sigma_{{e_{i} }}$” are the mean and standard deviation of the error for a given time “i” respectively.

The Nash–Sutcliffe Efficiency (NSE) in Eq. (24) that quantifies how well a model’s simulated values match the observed data. It essentially compares the residual variance (the difference between the model’s predictions and the actual observations) to the variance of the observed data itself. It’s important to note that NSE can be sensitive to outliers in the data^74^.

Where;

- NSE $\cong$ 1: Indicates a perfect match between the model and the observed data. This is the ideal scenario.
- NSE = 0: Suggests that the model’s predictions are no better than simply using the average of the observed data.
- NSE < 0: Implies that the observed mean provides a better prediction than the model. This indicates poor model performance.
- **Unsatisfactory prediction:** NSE ≤ 0.40, **Regular prediction:** 0.40 < NSE ≤ 0.60, **Good prediction:** 0.60 < NSE ≤ 0.80, **Excellent prediction:** 0.80 < NSE ≤ 1.00^74^.
  24 $$NSE = 1 - { }\frac{{\mathop \sum \nolimits_{{{\text{i}} = 1}}^{n} { }\left( {y_{{\text{i}}} \left( t \right) - z_{{\text{i}}} \left( t \right)} \right)^{2} }}{{\mathop \sum \nolimits_{{{\text{i}} = 1}}^{n} { }\left( {y_{{\text{i}}} \left( t \right) - {\overline{\text{z}}}_{{\text{i}}} \left( t \right)} \right)^{2} }}{ }$$

Where the actual value is $y_{{\text{i}}} \left( t \right)$, the forecasted value is $z_{{\text{i}}} \left( t \right)$, and the mean value of the forecasted value is ${\overline{\text{z}}}_{{\text{i}}} \left( t \right)$.

The determination coefficient R^2^ is the evaluation standard of the forecasting process using an artificial intelligence algorithm. The precision of the model increases as R^2^ approaches 1. The coefficient of determination, or R^2^, is a figure that expresses how well the data fits the regression model and can be either equal to or less than 1. It ranges from 0 (when there is no connection—poor correlation) to 1 (when the regression line crosses through all the data)^74^. The following Eq. (25) can express the determination coefficient R^2^.

25 $$R^{2} = 1 - \frac{{Sum of Squared Residuals \left( {SSR} \right)}}{{Total Sum of Squares \left( {SST} \right)}} = 1 - { }\frac{{\mathop \sum \nolimits_{{{\text{i}} = 1}}^{n} { }\left( {y_{{\text{i}}} \left( t \right) - z_{{\text{i}}} \left( t \right)} \right)^{2} }}{{\mathop \sum \nolimits_{{{\text{i}} = 1}}^{n} { }\left( {y_{{\text{i}}} \left( t \right) - {\overline{\text{z}}}_{{\text{i}}} \left( t \right)} \right)^{2} }}{ }$$

where the actual value is $y_{{\text{i}}} \left( t \right)$, the forecasted value is $z_{{\text{i}}} \left( t \right)$, and the mean value of the forecasted value is ${\overline{\text{z}}}_{{\text{i}}} \left( t \right)$.

#### Emulation of the 1st scenario’s outcomes

**Fig. 7:**
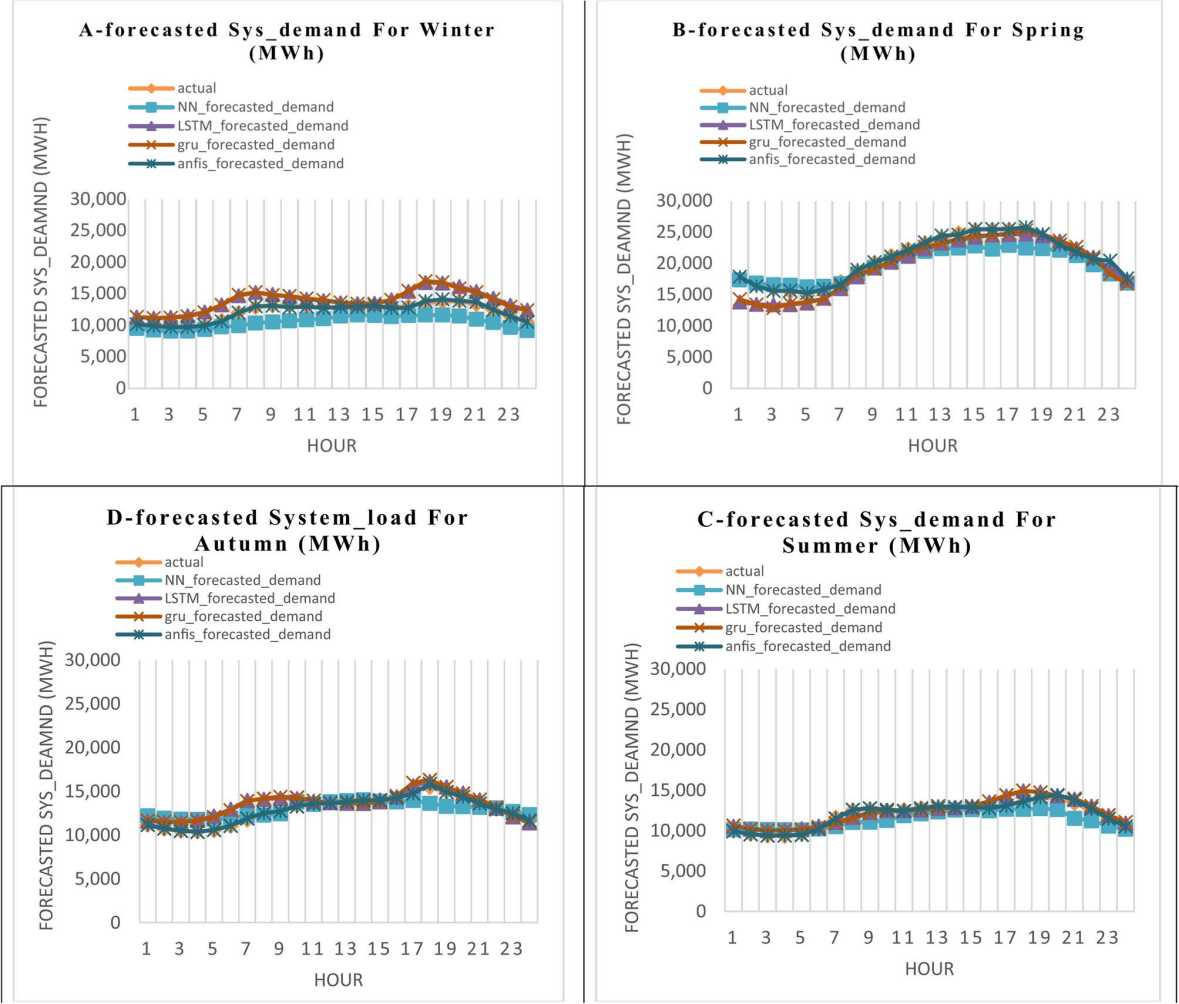
The actual and the forecasted results in the 1st. Scenario (workdays) of the Sys_demand (MWh), throughout a 24-h period in each of the four seasons by the four featured algorithms.

**Fig. 8:**
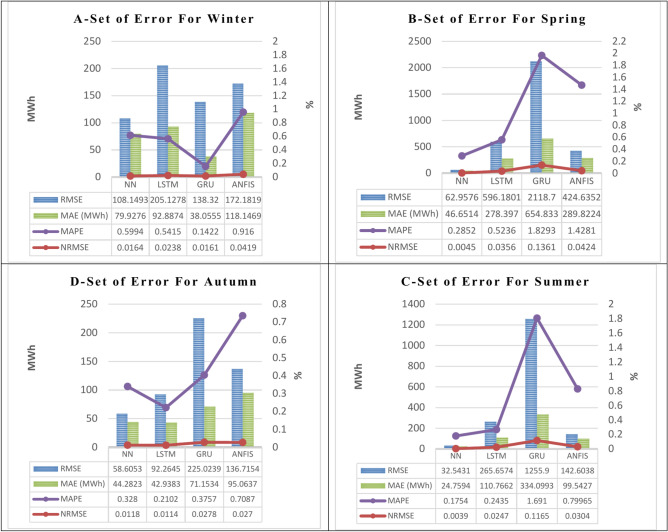
The calculated performance (RMSE, NRMSE, MAE, and MAPE) for the four specified algorithms in the four seasons for the Sys_demand (MWh), 1st. Scenario (workdays).

**Fig. 9:**
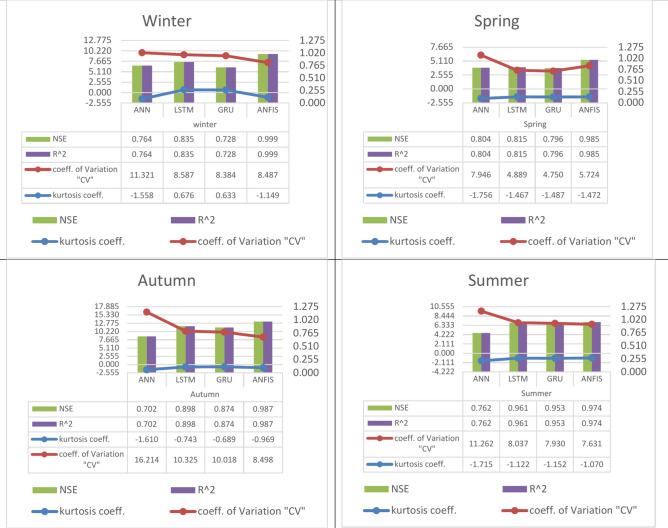
The calculated performance (The Nash–Sutcliffe Efficiency (NSE), the determination factor (R2), kurtosis coefficient, and coefficient of variation (CV)) for the four specified algorithms in the four seasons for the Sys_demand (MWh), 1st. Scenario (workdays).

The 1st scenario takes into account the prediction of the hourly energy load consumption (Sys_demand) of a workday depending on other factors mentioned in Table 1. The results for the 24 h of the final day of each season—March for winter, June for spring, September for summer, and December for autumn—are displayed in Fig. 7. These days are regarded as workdays. Figure 7 compares the actual and forecasted values of the hourly energy load consumption (Sys_demand) in (MW), which are simulated by the four algorithms ANN, LSTM, GRU, and ANFIS in the four seasons of winter, spring, summer, and autumn split into four sections in one figure A, B, C, and D, respectively. The set of errors (RMSE, NRMSE, MAE, and MAPE) for the dataset’s estimated values is shown in Fig. 8. The four suggested algorithms—ANN, LSTM, GRU, and ANFIS—simulate the outcomes, which are simulated in the four seasons. Figure 9 shows the calculated performance (The Nash–Sutcliffe Efficiency (NSE), the determination factor (R^2^), kurtosis coefficient, and coefficient of variation (CV)) for the four specified algorithms in the four seasons for the Sys_demand (MWh), 1st. Scenario (workdays).

#### Emulation of the 2nd scenario’s outcomes

**Fig. 10:**
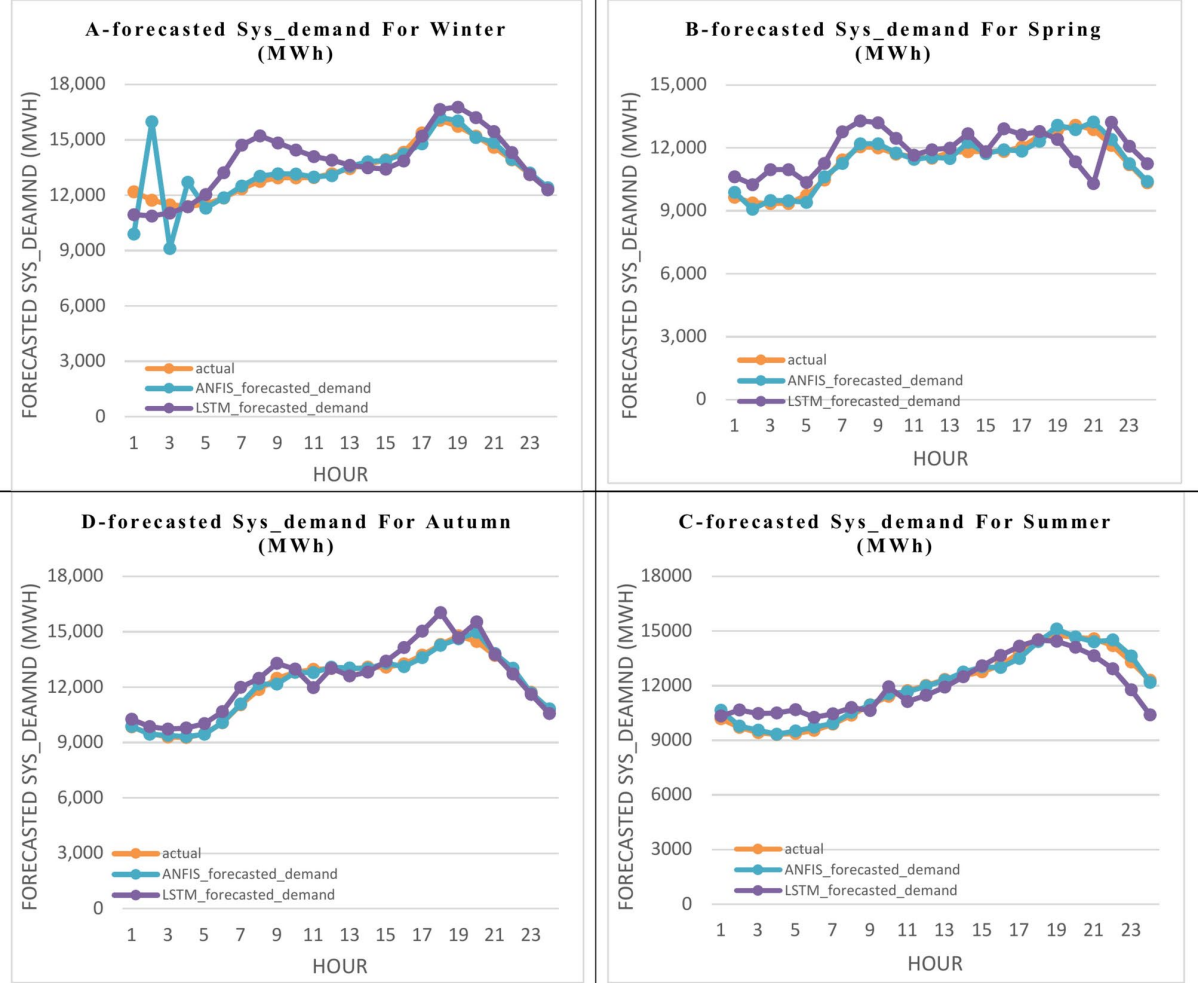
The actual and the forecasted results in the 2nd scenario (special holidays) of the Sys_demand (MWh), throughout a 24-h period in each of the four seasons by only two featured algorithms (GRU, and ANFIS).

**Fig. 11:**
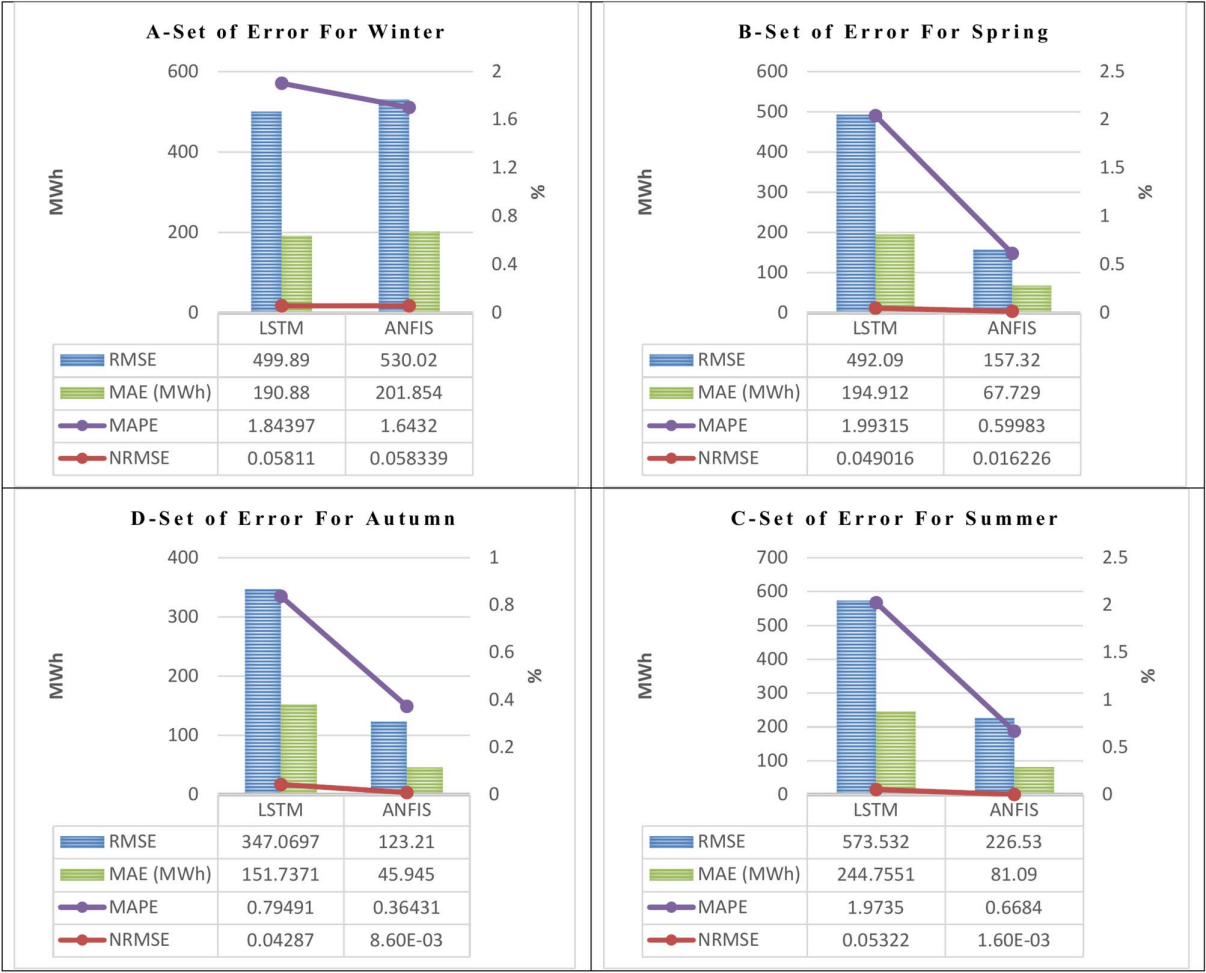
The calculated performance (RMSE, NRMSE, MAE, and MAPE) for the specified algorithms (LSTM, and ANFIS) in the four seasons for the Sys_demand (MWh), 2nd. Scenario (special holidays).

**Fig. 12:**
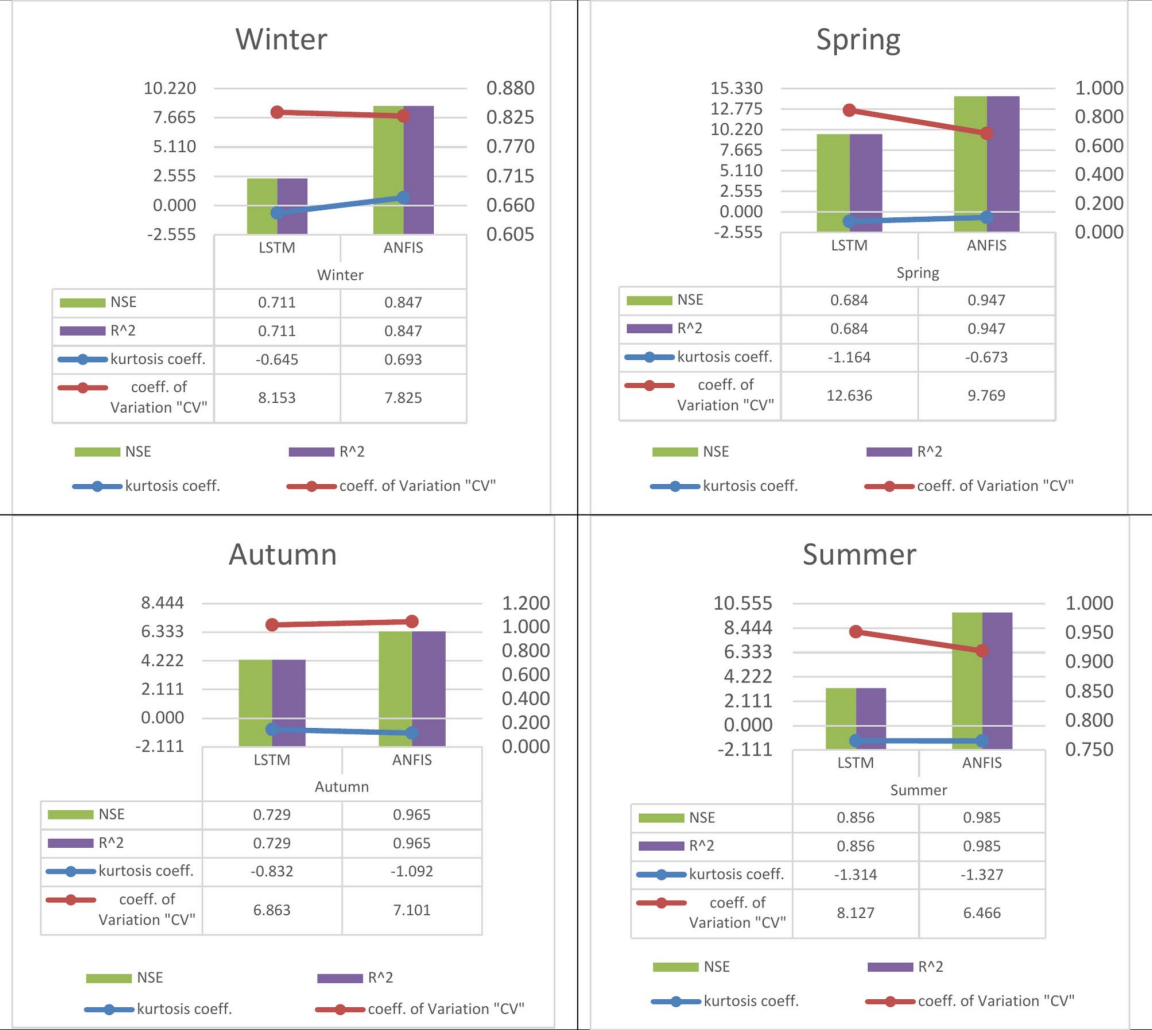
The calculated performance (NSE, R2, kurtosis coefficient, and coefficient of variation) for the specified algorithms (LSTM, and ANFIS) in the four seasons for the Sys_demand (MWh), 2nd. Scenario (special holidays).

The 2nd scenario takes into account the forecasting of the hourly load consumption (Sys_demand) of a weekend day that is considered a special holiday based on additional elements listed in Table 1. The results for the 24 h in each season—such as 1 January in winter which is New Year’s Day, 19 April in spring, which is Patriot’s Day, 5 July in summer which is Independence Day, and 11 October in autumn which is the Coulombs’ Day—are displayed in Fig. 10. These days are regarded as special holidays that may be followed by weekend days. Figure 10 compares the actual and predicted values of the hourly load consumption (Sys_demand) in (MW), which are modeled by using the LSTM, and the ANFIS algorithm in winter, spring, summer, and autumn split into four portions in one figure A, B, C, and D, respectively. The measured performance (RMSE, NRMSE, MAE, and MAPE) for the dataset’s estimated results is shown in Fig. 11. Considering that the LSTM and the ANFIS propose the best solution in the first scenario, they are chosen to simulate the set values of the error, which are obtained in the winter, spring, summer, and fall. Figure 12 shows the calculated performance (NSE, R^2^, kurtosis coefficient, and coefficient of variation) for the specified algorithms (LSTM, and ANFIS) in the four seasons for the Sys_demand (MWh), 2nd^.^ Scenario (special holidays).

The following notes can be summarized to describe the results in Figs. 7, 8, 9, 10, 11, and 12:

- The ISO-NE electricity market, which is split into four seasons, is where the datasets for the hourly load consumption (Sys_demand) and the hourly Market_price of electricity are collected.
- There are two scenarios for predicting the hourly load consumption (Sys_demand). The 1st^.^ Scenario forecasts the hourly energy load consumption of electricity (Sys_demand), in the working days depending on other parameters that are listed in Table 1 in the four seasons which are simulated by the four recommended techniques (ANN, LSTM, GRU, and ANFIS). In the 2nd^.^ Scenario, the hourly load consumption of electricity (Sys_demand), in the special holidays depending on other variables that are described in Table 1 by using the four seasons which is simulated by two of the four recommended techniques (LSTM, and ANFIS) that prove the best Nash–Sutcliffe Efficiency (NSE), determination factor (R^2^), kurtosis coefficient, and coefficient of variation (CV) in simulation in the 1st^.^ Scenario. The Nash–Sutcliffe Efficiency (NSE), the determination factor (R^2^), kurtosis coefficient, and coefficient of variation (CV) results in the two scenarios are:

A. In the 1st scenario, the Nash–Sutcliffe Efficiency (NSE), and the determination factor (R^2^) are (0.764, 0.835, 0.728, 0.999) for the winter, (0.804, 0.815, 0.796, 0.985) for the spring, (0.762, 0.961, 0.953, 0.974) for the summer, and (0.702, 0.898, 0.874, 0.987) for the autumn, modeled by the four featured algorithms (ANN, LSTM, GRU, and ANFIS). Hence, the LSTM as a machine learning technique and the ANFIS as a deep learning technique have proved the best Nash–Sutcliffe Efficiency (NSE), and determination coefficient values with excellent prediction rather than the other used techniques.
B. In the 1st scenario, kurtosis coefficient, and coefficient of variation (CV) are (− 1.558, 0.676, 0.633, − 1.149), and (11.321, 8.587, 8.384, 8.487) for the winter respectively, (− 1.756, − 1.467, − 1.487, − 1.472), and (7.946, 4.889, 4.750, 5.724) for the spring respectively, (− 1.715, − 1.122, − 1.152, − 1.070), and (11.262, 8.037, 7.930, 7.631) for the summer respectively, and (− 1.610, − 0.743, − 0.689, − 0.969), and (16.214, 10.325, 10.018, 8.498) for the autumn respectively, modeled by the four featured algorithms (ANN, LSTM, GRU, and ANFIS). It is obviously noted from the negative signs of the kurtosis coefficient Distributions with low kurtosis are referred to as platykurtic. Compared to normal distribution, they feature flatter peaks and lighter tails. This indicates that the probability of extreme readings is reduced.
C. In the 2nd scenario, the Nash–Sutcliffe Efficiency (NSE), and the determination factor (R^2^) are (0.711, 0.847) for the winter, (0.684, 0.947) for the spring, (0.856, 0.985) for the summer, and (0.729, 0.965) for the autumn, by two of the four featured techniques (LSTM, and ANFIS). Hence, the ANFIS as a deep learning technique have proved the best Nash–Sutcliffe Efficiency (NSE), and determination coefficient values with excellent prediction rather than the LSTM as a machine learning technique.
D. In the 2nd scenario, kurtosis coefficient, and coefficient of variation (CV) are (-0.645, 0.693), and ( 8.153, 7.825) for the winter respectively, (-1.164, -0.673), and (12.636, 9.769) for the spring respectively, (− 1.314, − 1.327), and (8.127, 6.466) for the summer respectively, and (− 0.832, − 1.092), and (6.863, 7.101) for the autumn respectively, modeled by two of the four featured techniques (LSTM, and ANFIS). It is obviously noted from the negative signs of the kurtosis coefficient Distributions with low kurtosis are referred to as platykurtic. Compared to normal distribution, they feature flatter peaks and lighter tails. This indicates that the probability of extreme readings is reduced.

- When forecasting the hourly energy load consumption (Sys_demand) in the 1st scenario that forecasts the working days by using the four techniques (ANN, LSTM, GRU, and ANFIS), Fig. 7 demonstrates that:

A. During the winter, the ANN’s predicted values and the real points match except for hours 8 through 11, and hours 19 through 21. The predicted results from LSTM and GRU progressively approach the real points except for hours 6 through 7, and 18 through 19. The ANFIS’s predicted results and the real points match very well.
B. During the spring, the ANN’s predicted results and the real points match except for hours 16 through 18. The predicted results from LSTM, and GRU progressively approach the real points except for hours 1 through 4. The ANFIS’s predicted results and the real points match very well.
C. During the summer, the ANN’s predicted results match the real points except for hours 20 through 22. Except for hour 18, the LSTM, GRUS, and ANFIS’s predicted results match the real points.
D. During the fall season, the results predicted by the ANN match the real points except for hour 18. The LSTM and GRU’s predicted results progressively approach the real values, except for hours 6 through 7. The ANFIS’s predicted values and the actual values match very well.

- Through a comparison of the performance parameters (RMSE, NRMSE, MAE, and MAPE) derived from the four proposed algorithms, in the two scenarios. It has been observed that:

1. Figure 8 in the 1st scenario forecasting model for workdays in the four seasons shows that:

A. During the winter, Gru’s performance (NRMSE, MAE, MAPE) is the best among the other techniques, which are decreased by (0.0003, 0.0077, 0.0258) for the NRMSE, (41.872, 54.832, 80.091) for the MAE, and (0.457, 0.399, 0.774) for the MAPE with respect to the same performance obtained from the ANN, LSTM, and GRU. However, the ANN’s performance in the RMSE is the best among the other techniques, which are decreased by (96.979, 30.171, 64.033) with respect to the same performance obtained from the LSTM, and GRU, ANFIS. In addition, the determination coefficient R^2^ indicates the best accuracy by simulating the results using the LSTM, and the ANFIS which are (0.835, and 0.999) for the winter.
B. During the spring, the ANN’s performance (NRMSE, RMSE, MAE, and MAPE) are the best among the other techniques, which are decreased by (533.223, 2055.742, 361.678) for the NRMSE, (0.031, 0.132, 0.038) for the RMSE, (231.746, 608.182, 243.171) for the MAE, and (0.238, 1.544, 1.143) for the MAPE with respect to the same performance obtained from the LSTM, GRU, and ANFIS. However, the determination coefficient R^2^ indicates the best accuracy by simulating the results using the LSTM, and the ANFIS which are (0.804, 0.815, 0.796, 0.985) for the spring.
C. During the summer, the ANN’s performance (NRMSE, RMSE, MAE, and MAPE) are the best among the other techniques, which are decreased by (233.114, 1223.357, 110.061) for the NRMSE, (0.021, 0.113, 0.027) for the RMSE, (86.007, 309.340, 74.783) for the MAE, and (0.068, 1.516, 0.624) for the MAPE with respect to the same performance obtained from the LSTM, GRU, and ANFIS. However, the determination coefficient R^2^ indicates the best accuracy by simulating the results using the LSTM, and the ANFIS which are (0.961, 0.974) for the summer.
D. During the autumn, the LSTM’s performance (NRMSE, MAE, MAPE) is the best among the other techniques, which are decreased by (0.0004, 0.0164, 0.0156) for the NRMSE, (1.344, 28.215, 52.125) for the MAE, and (0.118, 0.166, 0.499) for the MAPE with respect to the same performance obtained from the ANN, GRU, and ANFIS. However, the ANN’s performance in the RMSE is the best among the other techniques, which are decreased by (33.659, 166.418, 78.110) with respect to the same performance obtained from the LSTM, and GRU, ANFIS. In addition, the determination coefficient R^2^ indicates the best accuracy by simulating the results using the LSTM, and the ANFIS which are (0.898, 0.987) for the autumn.

2 Figure 11 in the 2nd scenario, which is simulated by one of the machine learning (LSTM) and one of the deep learning (ANFIS), for forecasting model of the special holidays (weekends) in the four seasons shows that:

A. During the winter, the LSTM’s performance (RMSE, NRMSE, MAE) is the best compared to the performance of the ANFIS, which is decreased by (30.130) for the RMSE, (0.00023) for the NRMSE, and (10.974) for the MAE with respect to the same performance obtained from the ANFIS. However, the ANFIS’s performance in the MAPE decreased by (0.2008) with respect to the same performance obtained from the LSTM. In addition, the determination coefficient R^2^ indicates the best accuracy by simulating the results using the LSTM, and the ANFIS demonstrates that the precision of the ANFIS is better than the LSTM which are (0.711, 0.847) for the winter.
B. During the spring, the ANFIS’s performance (RMSE, NRMSE, MAE, MAPE) is the best compared to the performance of the LSTM, which is decreased by (334.770) for the RMSE, (0.0328) for the NRMSE, (127.183) for the MAE, and (1.393) for the MAPE with respect to the same performance obtained from the ANFIS. In addition, the determination coefficient R^2^ indicates the best accuracy by simulating the results using the LSTM, and the ANFIS demonstrates that the precision of the ANFIS is better than the LSTM which are (0.684, 0.947) for the spring.
C. During the summer, the ANFIS’s performance (RMSE, NRMSE, MAE, MAPE) is the best compared to the performance of the LSTM, which is decreased by (347.002) for the RMSE, (0.0516) for the NRMSE, (163.6651) for the MAE, and (1.305) for the MAPE with respect to the same performance obtained from the ANFIS. In addition, the determination coefficient R^2^ indicates the best accuracy by simulating the results using the LSTM, and the ANFIS demonstrates that the precision of the ANFIS is better than the LSTM which are (0.856, 0.985) for the summer.
D. During the autumn, the ANFIS’s performance (RMSE, NRMSE, MAE, MAPE) is the best compared to the performance of the LSTM, which is decreased by (223.860) for the RMSE, (0.0343) for the NRMSE, (105.792) for the MAE, and (0.431) for the MAPE with respect to the same performance obtained from the ANFIS. In addition, the determination coefficient R^2^ indicates the best accuracy by simulating the results using the LSTM, and the ANFIS demonstrates that the precision of the ANFIS is better than the LSTM which are (0.729, 0.965) for the autumn.

## Discussion and the benefits of the provided study

It is evident from the literature reviewed in previous sections just how challenging it is to set up a load forecasting mechanism. Notwithstanding variations in the choice of the input variable, scope prediction, preprocessing to be used, method selection, parameter estimate, and performance assessments, a few recommendations to assist the novice developer have been recorded. The primary factors that our study has attempted to implement and address when building a load/price-forecasting challenge are as follows:

- One of the biggest oscillations and the root causes of daily price changes is the features of the electrical markets under the effect of the temperature and the calendar (workdays, and weekends).
- To obtain more accurate predictions, large records of the hourly load and price are necessary due to the short-term inelasticity of power demand among the seasons.
- Additional characteristics, such as weather parameters (dew point and dry bulb), humidity, day type, hour-by-hour load, price, and GDP, are taken into account in the forecasting model. These are intrinsic factors that affect the prediction of either the load or the price.
- The requirement for more advanced and reliable algorithms that can forecast non-linear and inelastic data and price, such as neural-based networks.
- The suggested algorithms’ evaluation performance showed that the best solutions had the fewest group of errors (RMSE, NRMSE, MAE, and MAPE), with a MAPE of no more than 6%. High accuracy has also been attained during unexpected surges in the load/price patterns under the effect of the temperature and the calendar (workdays, and weekends).

### Temperature effects

Many studies have examined the impact of climate variables, particularly temperature, and short-term demand forecasting models take this into account. Geographical diversity has a significant cooling influence in warm nations and a significant heating effect in cold countries^15,19,43,50,51,75^.

Wintertime temperatures in New England can range from mild to bitterly cold, and as one might expect, the further north you go in the state, the lower the average annual temperature where the average winter temperature is − 6.111 degrees Celsius. For this reason, heating equipment is widely used in NEW England. In the Northeast, the average June, July, and August temperatures are (29.444 to 32.222) degrees Celsius (including overnight lows). This highest range of average temperatures encourages utilizing cooling appliances like air conditioners. The difference in demand between non-holiday and holiday periods at peak hours (6 p.m.) in winter, (8 p.m.) in spring, (2 p.m.) in summer, and (7 p.m.) in autumn owing to temperature. The temperature has a discernible effect on peak demand. During working days, there is a sharp and linear demand for electricity. A large fluctuation is considered in load patterns on holidays when the weather falls beneath 30 °C or ascends above 35 °C.

### Calendar effects

Demand for electricity exhibits a recurring pattern that is based on weekly, monthly, and seasonal patterns. However, variables like temperature or social gatherings frequently throw off these trends and introduce unpredictable outlier data. In order to categorize exceptional days without requiring any prior database knowledge, it is insufficient to simply divide special days into two or three categories because there are various nuances in customer behavior on these days. However, categorizing special days into a broad variety of groups necessitated a thorough comprehension of how customers behaved on various days and throughout the year^76,77^.

Calendar effects have a very predictive value for the hourly demand consumption prediction, according to the metric performance results (RMSE, NRMSE, MAE, MAPE). When compared to the first scenario which forecasts the workdays with the second scenario which forecasts special holidays, the prediction models using dummy variables demonstrated a consistent forecast improvement when using many dummy variables in the forecasting model. Adding more dummy variables to the forecast for weekends, and weekdays in different seasons did help. The somewhat increased accuracy or the determination factor R^2^ when all calendar effects are taken into account. Calendar effects are such as national holidays, days adjacent to a holiday, partial holidays, common vacation periods, and other calendar factors that cause the load profile of a day to change drastically. Including all calendar effects may result in a significant improvement in the metric performance (NRMSE, and MAPE) in addition to the determination factor R^2^. Results from dividing by day and time of day were significantly better than those from dividing by season. Although groups with more distinct load and weather characteristics are created when sequential input data is joined into subsets based on similar calendar effects, The swings in demand at peak times resulted in greater RMSE and NRMSE.

Therefore, potential limitations that we could conclude from this study in the future, and areas for improvement:

1. Data limitations:
  The need for larger datasets spanning multiple years is emphasized, as consumer behavior and consumption patterns can change over time.
  Incorporating sociodemographic factors into the analysis could help account for these shifts.
2. Day type classification:
  The importance of accurately classifying different types of day (weekdays, weekends, holidays, etc.) is highlighted to be built in a special forecasting network.
  A technique for automating the classification process based on historical data and calendar factors is suggested.
3. Algorithm enhancement:
  Combining existing techniques like ANFIS, AGA-LSTM, and LSTM-RNN with other machine and deep learning methods is proposed to achieve even lower forecasting errors (MAPE).

## Conclusion

It became obvious that developing a forecasting model was considered the main way of addressing the sharp rise in nonlinear load patterns and power price dynamics. ANN, LSTM, GRU, and ANFIS were proposed in this study as four prominent ways that were suggested to increase forecasting accuracy and speed with a collection of mistakes (RMSE, NRMSE, MAE, and MAPE). The ISO-NE electrical power energy market was the source of the complete dataset for hourly demand consumption (Sys_demand), hourly Market price, and other factors. Where the datasets spanned the period of January 1, 2021, to December 31, 2021, or one year. The four seasons—winter, spring, summer, and autumn—were represented by the dataset divisions. Following preprocessing and analysis of both the hourly demand consumption (Sys_demand) and Market_price datasets, the four featured algorithms in each of the first scenarios, and two of the best of the four featured algorithms were implemented in the second scenario. Where the first one was to forecast the workdays and the second one was to forecast the special holidays as an example of weekend days. The two scenarios were divided into four seasons. The forecasting model has been affected mainly by calendar, temperature, and seasonality.

A set of errors was used in assessing the four main algorithms. By comparing the findings from the two scenarios, it has become apparent that ANFIS in the summer and autumn in the 1st scenario, and in the spring and autumn in the 2nd scenario, has provided the best-predicting performance with the least amount of MAPE. The best forecasting performance (MAPE) was provided by GRU in the winter in the first scenario when MAPE was at its lowest value (0.1422). The best forecasting performance (MAPE) was provided by ANFIS in the autumn in the second scenario when MAPE was at its lowest value (0.3643). However, for the two scenarios, it was found that the best forecasting accuracy with the lowest MAPE was provided by LSTM and ANFIS.

By comparing the accuracy from the two scenarios, it has become apparent that ANFIS in the 4 seasons in the 1st scenario, and the 2nd scenario except in the winter, has provided the highest accuracy. Where the determination factor R^2^ has a minimum value (97.40%) in the summer and a maximum value (99.90%) in the winter for the 1st scenario. For the 2nd scenario, the determination factor R^2^ has a minimum value (98.50%) in the summer and a maximum value (84.70%) in the winter.

For simulating the results by LSTM, by comparing the accuracy from the two scenarios. It is found that the determination factor R^2^ has a minimum value (81.50%) in the spring and a maximum value (96.10%) in the summer for the 1st scenario. For the 2nd scenario, the determination factor R^2^ has a minimum value (68.40%) in the spring and a maximum value (85.60%) in the winter.

The majority of demand consumption forecasting studies used the impact of calendar and load data profiles from past records as self-explanatory factors. This manuscript has shown how different seasonal effects, and temperature, calendar effects, hourly price, hourly demand itself affected the precision of the hourly short-term load forecast for one day while taking different predicting algorithms into consideration. We showed that the dividing approach into seasons and types of days according to the calendar has had a higher forecast accuracy because more training data would increase the precision of forecast models. In this study, the calendar effects that were included as binary variables produced a smaller inaccuracy. To reduce the amount of time and computing resources needed, only significant predictors should be included in forecasting models. Such as calendar, seasonal, and temperature effects, hourly price and demand need inside data, this study has demonstrated that they have become more significant in the forecasting model. Since the LSTM as one of the machine learning algorithms, and the ANFIS as one of the deep learning algorithms achieve the best in accuracy or determination factor R^2^ and the performance of the measured performance (RMSE, NRMSE, MAE, and MAPE) in simulating the first scenario, they are used in the second scenario.

However, the characteristics of the electrical markets are one of the main oscillations and the main drivers of daily price fluctuations. Because of the seasonal variations in the short-term inelasticity of power demand, extensive hourly load and price records are required to produce more precise forecasts. The forecasting model also considers other elements including humidity, day type, hourly load, price, GDP, and meteorological parameters like dew point and dry bulb. These are inherent factors that influence the load or pricing projection.

This work could lead to the conclusion that several issues might need to be investigated further in future research:

- One could argue that this could be resolved by having the ability to make use of a bigger dataset. The issue with data from multiple years indicates that there typically are different and various lifestyles of the consumers, and the population or consumption pattern may alter over time. Then, adding sociodemographic factors may aid in addressing these shifts.
- A technique suggests an algorithm to automate branching into as many categories as necessary to match an actual and a large database for many years, starting with a basic day-of-the-week classification. Classification of the type of the day is necessary, especially for national holidays, days adjacent to a holiday, partial holidays, common vacation periods, and other calendar factors that cause the load profile of a day to change unexpectedly.
- To get forecasted values with the lowest MAPE, it is required to combine existing techniques—like the hybridized ANFIS, the adaptive genetic algorithm (AGA-LSTM), and the integration of LSTM and recurrent neural network (RNN)—with additional machine and deep learning techniques.

## Data Availability

All data generated or analysed during this study are included in this manuscript [References in the manuscript:^70^ Independent System Operator New England (ISO-NE) https://www.iso-ne.com, (accessed on: 7 October 2023)^71^.Index of /Data. Available online: https://www.timeanddate.com/weather/@7,288,047/climate/, (accessed on: 2 August 2022). ].

## Abbreviations

GRU: Gated recurrent units
LSTM: Long short-term memory
ANFIS: Adaptive neuro-fuzzy inference system
FIS: Fuzzy inference system
ANN: Artificial neural network
RNN: Recurrent neural network
$\check{C}_{t}$: The cell’s state of LSTM
$F_{t}$: The forget gate of LSTM
$i_{t}$: The input gate of LSTM
$\overline{{\mathop {\text{O}}\limits^{.} }}_{t}$: The output gate of LSTM
$x_{t}$: The input signal of GRU
$h_{t}$ and $h_{t}^{ - }$: The transient outcome and the hidden layer output, at moment t in the GRU algorithm
$Z_{t}$ and $R_{t}$: The variables’ outcome of the modify and reset gates at t, respectively.
(X) and tanh(X): The sigmoid and tanh activation functions, respectively.
$a_{{\text{i}}}$, $b_{{\text{i}}}$ and $c_{{\text{i}}}$: The arrangement of elements that are determined throughout the model-learning process in the ANFIS.
$R_{{\text{i}}}$ and $S_{{\text{i}}}$: The fuzzy sets in ANFIS
$F_{{\text{i }}}$: The output of ANFIS
${\text{x}}_{1}$ and ${\text{x}}_{2}$: The inputs of ANFIS
$$\hat{C}_{i}$$: The candidate which is scaled by $$\underset{\raise0.3em\hbox{$\smash{\scriptscriptstyle\cdot}$}}{\text{i}}_{t}$$ in LSTM and GRU structure
N: Number of samples
$\hat{y},{ }$ y: The forecasted value and the actual value of the load respectively.
SSE: Sum squared error
MSE: Mean squared error
RMSE: Root mean square error
MAE: Mean absolute error
MAPE: Mean absolute percentage error
